# An Explainable Deep Learning-Based Predictive Maintenance Solution for Air Compressor Condition Monitoring

**DOI:** 10.3390/s25185797

**Published:** 2025-09-17

**Authors:** Alexandru Ciobotaru, Cosmina Corches, Dan Gota, Liviu Miclea

**Affiliations:** Automation Department, Faculty of Automation and Computer Science, Technical University of Cluj-Napoca, 400114 Cluj-Napoca, Romania; alexandru.ciobotaru@aut.utcluj.ro (A.C.); cosmina.corches@aut.utcluj.ro (C.C.); liviu.miclea@aut.utcluj.ro (L.M.)

**Keywords:** predictive maintenance, condition monitoring, deep learning, SVM air compressor, SHAP, LIME, PDP

## Abstract

Air compressors are vital across various sectors—automotive, manufacturing, buildings, and healthcare—as they provide pressurized air for air suspension systems in vehicles, supply power pneumatic machines throughout industrial production lines, and support non-clinical infrastructure within hospital environments, including pneumatic control systems, isolation room pressurization, and laboratory equipment operation. Ensuring that such components are reliable is critical, as unexpected failures can disrupt facility functions and compromise patient safety. Predictive maintenance (PdM) has emerged as a key factor in enhancing the reliability and operational efficiency of medical devices by leveraging sensor data and artificial intelligence (AI)-based algorithms to detect component degradation before functional failures occur. In this paper, a predictive maintenance solution for condition monitoring and fault prediction for the exhaust valve, bearings, water pump, and radiator of an air compressor is presented, by comparing a hybrid deep neural network (DNN) as a feature extractor and a support vector machine (SVM) for condition classification: a pure DNN classifier as well as a standalone SVM model. Additionally, each model was trained and validated on three devices—NVIDIA T4 GPU, Raspberry Pi 4 Model B, and NVIDIA Jetson Nano—and performance reports in terms of latency, energy consumption, and CO_2_ emissions are presented. Moreover, three model agnostic explainable AI (XAI) methods were employed to increase the transparency of the hybrid model’s final decision: Shapley additive explanations (SHAP), local interpretable model-agnostic explanations (LIME) and partial dependence plots (PDP). The hybrid model achieves on average 98.71%, 99.25%, 98.78%, and 99.01% performance in terms of accuracy, precision, recall, and F1-score across all devices Additionally, the DNN baseline and SVM model achieve on average 93.2%, 88.33%, 90.45%, and 89.37%, as well as 93.34%, 88.11%, 95. 41%, and 91.62% performance in terms of accuracy, precision, recall, and F1-score across all devices. The integration of XAI methods within the PdM pipeline offers enhanced transparency, interpretability, and trustworthiness of predictive outcomes, thereby facilitating informed decision-making among maintenance personnel.

## 1. Introduction

**Figure 1:**
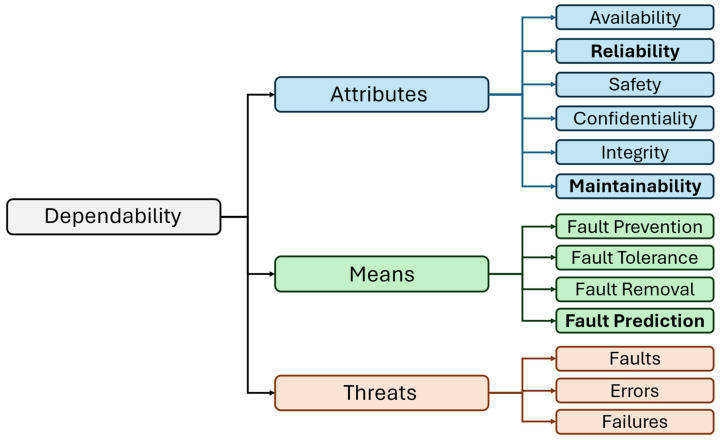
Dependability tree overview with highlighted attributes and addressed means.

In contemporary industrial and infrastructural applications, air compressors increasingly operate as cyber-physical systems (CPS), combining physical processes with integrated processing, sensing, and AI-based algorithms. As they often serve as backbone components in automation, pneumatic control, and environmental regulation, enhancing the dependability of such systems is critical across various industrial sectors. This becomes especially relevant in various domains such as automotive, manufacturing, or healthcare, where air compressors support key systems such as air suspension, air brakes, and various pneumatic tools (e.g., impact wrenches, drills, sanders, grinders, air hammers, etc.) or non-clinical systems such as isolation room pressurization, laboratory operations, and medical utility controls. Additionally, air compressors enable heating, ventilation, and air conditioning (HVAC) operations in building environments by maintaining optimal indoor temperatures. Therefore, any failure or unplanned downtime in these systems can compromise operational continuity in factories, delay medical procedures, or even pose safety risks. As shown in Figure 1, ensuring key dependability attributes such as reliability and maintainability, by means of fault forecasting using air compressors predictive monitoring, is essential for maintaining performance, reducing risk, and supporting operations in automotive, manufacturing, and hospital environments.

Predictive maintenance (PdM) offers a significant advancement in the healthcare sector over traditional preventive and reactive maintenance strategies. It enables condition-based interventions, rather than relying on fixed schedules or post-failure responses [1] and thus, enhances CPS maintainability. Unlike reactive maintenance, which incurs unexpected downtime and potential safety risks due to equipment failure, PdM anticipate failures before they occur through real-time monitoring and data-driven diagnostics. In addition, compared to preventive maintenance, where components are replaced or serviced at regular intervals regardless of actual wear, PdM minimizes unnecessary maintenance actions, reduces operational costs, and extends the useful life of equipment [2,3]. This approach is particularly valuable in critical environments such as hospitals, where maintaining continuous system functionality is essential; unplanned outages can compromise patient care or disrupt facility operations [4]. Additionally, according to Grand View research [5], the US PdM market was estimated at 7.85 billion dollars in 2022 and is estimated to be worth approximately 60.13 billion dollars by 2030, due to significant advances in AI systems. Moreover, condition monitoring represents a key element for efficient fault diagnosis not only in the healthcare sector, but also in the context of intelligent transportation (i.e., using overspeed information monitoring) [6] or in the aerospace domain (i.e., via aero-engine pipeline monitoring using a triboelectric-piezoelectric clamp with self-sensing capability) [7]. Table 1 presents a comparison of this study and related work in the area of PdM solutions for air compressors using machine learning (ML) and DL methods from both supervised and unsupervised points of view.

Recent studies have presented numerous ML- and DL-based PdM solutions for air compressor condition monitoring in a wide variety of environments. The study conducted by Panda C. et al. [8] focused on reducing commercial vehicle downtime via ML-based PdM, using air compressor failure in long-distance trucks as a case study. The authors compare three decision tree models (i.e., CART, C5.0, and C5.0 with boosting), achieving 95% specificity and 76% recall. Based on the truck’s service record, the air compressor is labeled as faulty or non-faulty. In the study conducted by Daoudi N. [9], four supervised ML-based models were utilized to predict the outlet temperature of the screw element from an industrial GA132 screw compressor based on time series data, as well as the compressor outlet pressure or ambient air temperature. Similarly, the study conducted by Zanoli, S. et al. [10] presents a practical implementation of a PdM algorithm of a twin screw oil-injected air compressor using supervised ML-based models such as Fine Tree or Ensemble Bagged Trees, which can differentiate between four maintenance urgency classes (i.e., long, medium, short and urgent). Additionally, a total of 12 features (i.e., motor current, air flow rate, air pressure, pulled air temperature, oil temperature, etc.) were utilized to identify four maintenance conditions, reflecting the required intervention severity and urgency. A study conducted by Aminzadeh A. et al. [11] proposed an IoT-integrated PdM system for industrial air compressors via linear regression; they presented a lightweight ML-based model for predicting overheating risk and motor stress. Furthermore, for real-time data acquisition, Siemens PLCs were utilized. Moreover, Barpute J. et al. [12] proposed a semi-supervised ML-based PdM algorithm for air compressors in metro train systems, using the MetroPT-3 dataset. The authors use the isolation forest classifier to detect anomalies and classify them as failures. The next step is the anomaly detection phase, using five ML-based algorithms such as logistic regression, random forest, and XGBoost. Notably, XGBoost obtained the best results (i.e., over 99% accuracy) while logistic regression obtained the lowest training time (i.e., 13 s). Another complex PdM-based air compressor radiator condition monitoring and fault prediction method was presented in [13]. The authors proposed a hybrid DL-based approach, whereby the motor power consumption over time is predicted using Long Short-Term Memory (LSTM) models. Moreover, radiator condition (i.e., dirty or clean) was predicted based on various air compressor features, such as RPM, water pump power, oil tank temperature, etc., using various ML-based models (i.e., logistic regression, random forest, SVM, XGBoost, etc.). Therefore, the proposed hybrid approach achieved 93% accuracy in radiator fault detection. The study conducted by Farid A. et al. [14] proposes a PdM solution for a screw air compressor, focusing on bearing and gear faults detection via vibration analysis. By applying spectral and envelope techniques (i.e., Fourier and Hilbert transforms), the system identifies early-stage degradation and estimates the remaining useful life, thereby enabling timely intervention. Recently, numerous studies have integrated various XAI techniques, highlighting the reasoning behind DL models. For instance, M. Jiang et al. [15] proposed an explainable and generalizable fault diagnosis method for metro train air conditioning systems, using an XGBoost model combined with SHAP for interpretability. It addresses three single faults (i.e., condenser fouling, ventilation fouling, and refrigerant leak). The model achieves competitive performance (i.e., accuracy increased by up to 8.38%, and false alarm rates dropped by over 11%) using a selected subset of features, with key contributors identified as evaporator outlet enthalpy, condenser outlet temperature, and airflow rate. Additionally, SHAP analysis provides insight into feature influence on both single and simultaneous fault predictions, enhancing model transparency. S. M. Farea et al. [16] proposed an interpretable machine learning method for detecting failures in air pressure systems (APSs) of heavy-duty vehicles using the Explainable Boosting Machine, a glass-box model offering strong predictive accuracy and intrinsic explainability. Using real-world data from 110 healthy and 30 faulty vehicles, the model achieved a classification accuracy of 91.4%, an F1 score of 0.80, and an AUC of 0.88 through stratified five-fold cross-validation. Additionally, XAI-enhanced DL models have been widely utilized for fault prediction and condition monitoring in HVAC systems. For instance, M. Meas et al. [17] proposed an explainable fault detection system for air-handling units using an XGBoost classifier combined with SHAP to identify and explain five common HVAC faults (e.g., sensor failures, valve leaks, heat recovery issues) from real-world building data. The model achieves high performance, with a weighted F1 score of 99.7% and accuracy of 99.6%, outperforming random forest and logistic regression. Moreover, the authors of [18] presented a two-stage fault detection and diagnosis framework for air-handling units using XGBoost classifiers enhanced with SHAP for interpretability. Real-world data from a commercial building, spanning a year, were used to train models to identify the normal state and four specific fault types: sensor malfunction, heat recovery failure, heating coil valve leakage, and cooling coil controller issues. The system achieves high classification performance, with XGBoost reaching an F1 Score of 97% for fault detection, and between 90% and 99% for individual fault types in diagnosis. Additionally, the study conducted by K. Chen et al. [19] presents an interpretable DL framework for fault diagnosis in HVAC systems, using a 1D-Convolutional Neural Network enhanced with the Score-CAM method for explainability. N. Es-Sakali et al. [20] proposed a cloud-based system for detecting refrigerant leaks in HVAC systems using four machine learning models, notably random forest, achieving an accuracy of up to 99.3%. Trained on fault data collected from a single-compressor screw chiller, the model achieved 97.5% accuracy in classifying three fault types: refrigerant leakage, reduced water flow in the condenser, and reduced water flow in the evaporator. A. Nambiar et al. [21] proposed a fault diagnosis system for single-stage air compressors using vibration signal analysis, feature fusion and ML-based algorithms (i.e., KNN, local KNN or random subspace ensemble KNN). Vibration data were collected under four fault types (inlet valve fluttering, outlet valve fluttering, combined inlet-outlet fluttering, and check valve fault). Additionally, the fusion between three feature types (i.e., auto-regressive moving average, statistical attributes, and histogram data), in conjunction with local KNN achieved an overall accuracy of 100%. Moreover, the authors of [22] introduced a deep copy stacked ensemble model (using, for example, logistic regression, Decision Trees, SVM, KNN, Naïve Bayes) which can detect various air compressor fault types (i.e., cooler, valve or air filter condition), achieving 99.3% accuracy, 96.7% precision, and 100% recall. Similarly, the authors of [23] compared 24 supervised learning algorithms for the predictive maintenance of a twin-screw oil-injected air compressor. The system categorizes four levels of maintenance urgency based on forced degradation scenarios. The optimizable ensemble model achieved the best performance with 99.7% accuracy and 95% recall. Apart from using supervised learning methods, unsupervised learning techniques were also employed for air compressors’ health management [24,25,26,27]. For instance, the authors of [24] stacked autoencoders, the system learns patterns from healthy compressor behavior and computes anomaly scores to detect deviations in sensor data, including vibration, pressure, temperature, and oil-related parameters. Additionally, S. M. Zanoli et al. [25] presented an unsupervised fault-detection approach using k-means clustering and principal component analysis to predict degradation in twin-screw air compressors. Instead of labeling explicit faults, the system categorizes compressor-operating states into long, medium, and urgent, representing the increasing severity of oil degradation and volume loss. These states simulate real-world lubrication issues and allow the model to assess compressor health based on multivariate sensor data (e.g., temperatures, pressures or motor current). Additionally, the authors of [26] developed a deep learning-based anomaly detection system for identifying bearing faults in an industrial air compressor, using an LSTM-Recurrent Neural Network architecture. The model is trained on two years of tri-axial vibration data, collected while operating an injection-molding machine. After converting time-domain data into frequency spectra using short time Fourier Transform, the model predicts machine states classified as stopped, normal, near failure, and failure. With a maximum accuracy of 97.4%, the LSTM model outperformed several baseline classifiers (e.g., SVM, Convolutional Neural Network or XGBoost). In [27], the authors integrate hybrid clustering techniques with classification models such as SVC, Random Forest, and KNN to improve fault detection in high-pressure industrial compressors, improving the detection accuracy by an average of 4.87%. Based on the studies presented in Table 1, most recent papers do not consider XAI techniques to highlight the internal decision-making process of the ML- or DL-based models, or utilize only one (i.e., mainly SHAP diagrams). While Shapley additive explanations (SHAP) diagrams offer valuable insights in terms of both feature importance and directionality, complementary methods such as local interpretable model-agnostic explanations (LIME) and partial dependence plots (PDP) highlight the model’s behavior at the level of a specific feature or show the average impact of a certain feature across the entire dataset (i.e., as the feature evolves, its overall condition is shown dynamically). In this way, actionable maintenance decisions are made both at the instance level and at the component level.

**Table 1:** Comparative analysis with related results.

Ref.	Domain	Analyzed Machine	ML/DL Technique	Performance	XAI Techniques
[8]	Transport Domain	Air compressor (truck)	CART, C5.0, C5.0, Gradient Boosting	Accuracy—86% Recall—87% Precision—91%	×
[12]		Metro train air compressor	Isolation Forest, Logistic Regression, RF, XGBoost, CatBoost, LightGBM	Accuracy—99.7% F1 Score—99.7%	×
[15]		Metro train AC system	XGBoost	Accuracy increase by 5.84–8.38%	SHAP
[16]		Air pressure system	Explainable Boosting Machine	Accuracy—91.4% F1-Score—80% AUC—0.88	Intrinsic (EBM)
[9]	Industrial Domain	Oil-injected screw compressor	Linear Regression, KNN, SVM, Gradient Boosting	MSE—17.94 RMSE—4.24 MAE—1.95 R2—0.92	×
[10]		Twin-screw oil-injected compressor	Optimizable Ensemble (e.g., boosted trees)	Accuracy—99.7%	×
[11]		General purpose industrial compressor	Linear Regression	Accuracy—98% Decrease in Downtime by 20%	×
[13]		Radiator in compressed air system	LSTM, Logistic Regression, RF, SVM, XGBoost, LightGBM	Accuracy—93% Energy savings by 2.24%	×
[14]		Air screw compressor	Spectral and envelope analysis (FFT + Hilbert), RMS	Effective early fault detection via vibration signals.	×
[21]		Single-stage air compressor	KNN, local KNN, locally weighted learning, random subspace ensemble KNN	Accuracy—100%	×
[22]		Single-stage air compressor	Stacked ensemble (Linear Regression, Decision Tree, SVM, KNN, Naïve Bayes)	Accuracy—99.3% Precision—96.7% Recall—100%	×
[23]		Twin-screw oil-injected air compressor	Ensemble-based model (24 classifiers)	Accuracy—99.7% Recall—95%	×
[25]		Twin-screw air compressor	Principal Component Analysis and K-Means Clustering	Accurate clustered degradation levels.	×
[27]		High-pressure industrial compressor	Hybrid Clustering and Classification (SVC, Random Forest, KNN)	Accuracy—97.9%	×
[17]	HVAC Systems	Air handling units	XGBoost	Accuracy—99.6% F1-Score—99.7%	SHAP
[18]		Air handling units	XGBoost	F1-Score—97%	SHAP
[19]		Screw chillers	1D-Convolutional Neural Network	Accuracy—80.27%	Score-CAM
[20]		Variable refrigerant flow	Decision Tree, Random Forest, KNN, SVM	Accuracy—99.3% Precision—99.4% Recall—99%	×
[24]	Manufacturing Domain	General purpose air compressors	Stacked autoencoders	Anomaly scores highly correlate with failure.	×
[26]		Twin-screw air compressor	LSTM-Recurrent Neural Network with hierarchical clustering	Accuracy—97.4%	×
This Paper *	**Industrial** **Domain**	**Single-stage, water-cooled air compressor**	**DNN + SVM**	**Accuracy—98.58%** **Precision—99.11%** **Recall—98.27%** **F1-Score—98.62%**	**SHAP, LIME, and PDP**
			**DNN**	**Accuracy—93.23%** **Precision—88.33%** **Recall—90.45%** **F1-Score—89.37%**	
			**SVM**	**Accuracy—93.34%** **Precision—88.11%** **Recall—95.41%** **F1-Score—91.62%**	

* Results obtained by the hybrid DL-based model, a two-layer DNN architecture, and a standalone SVM model.

The main contributions of this paper are as follows:

- A supervised PdM-based condition monitoring solution for the four principal components of an air compressor (i.e., the exhaust valve, bearings, water pump and radiator)
- A comparison between a hybrid deep learning (DL) model composed of a deep neural network (DNN) for feature extraction and support vector machines (SVM), a pure two-layer DNN model, and a standalone SVM model for fault classification (i.e., clean/dirty in the case of the exhaust valve and radiator and healthy/noisy in the case of the bearings and water pump).
- A comparison of the hybrid model performance on three devices: two general-purpose computing devices (i.e., a machine equipped with an NVIDIA T4 GPU and NVIDIA Jetson Nano) and one device with limited resources (i.e., Raspberry Pi 4 Model B) in terms of training and inference latency and energy consumption, as well as carbon oxide emissions.
- The utilization of three explainable AI (XAI) techniques that enhance the hybrid architecture’s transparency and interpretability: two global model agnostic methods (i.e., SHAP and PDP) and one local model agnostic method (i.e., LIME).
- A comparison in terms of performance and impact on XAI interpretability between the hybrid model, two-layer DNN baseline, and the standalone SVM model using SHAP diagrams.

The remainder of the paper is organized as follows. Section 2 presents the dataset, the device configuration used to train the DL-based models, a brief overview of the XAI methods used, and the performance metrics used to validate the DL-based model. Section 3 highlights the experimental results after validating the models on all devices, the obtained SHAP, LIME and PDP diagrams, and the performance reports across all devices. Finally, Section 4 concludes the study and presents possible future research directions.

## 2. Methodology

**Figure 2:**
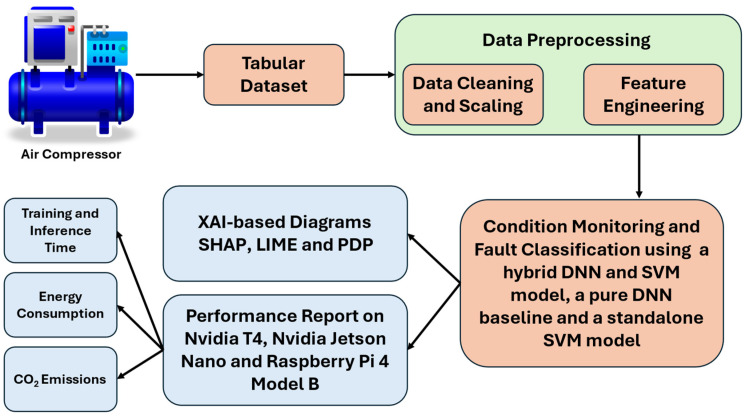
Overview of the proposed methodology.

This section presents the proposed methodology used to develop the PdM algorithm for air compressor condition monitoring and fault classification. An overview of the overall methodology is presented in Figure 2. In fact, an exploratory data analysis (EDA) description of the tabular data is outlined in this section, which also includes the preprocessing steps, as well as the structure of the developed DL-based model. Additionally, we present the included XAI techniques to provide a visual understanding of the predictions made by the hybrid DL model. Moreover, the hybrid DL-based model was trained and validated on three device types: two general purpose devices (i.e., NVIDIA T4 GPU and NVIDIA Jetson Nano) and two devices with limited resources (i.e., Raspberry PI 4 Model B).

### 2.1. Exploratory Data Analysis of the Air Compressor Dataset

Exploratory data analysis (EDA) represents an important initial step in developing a robust DL-based model, particularly when working with tabular-based datasets. Unlike image-based datasets, which often benefit from visual intuition, tabular-based datasets require careful inspection to uncover relationships, anomalies, and patterns hidden in both the dependent and independent variables. In addition, for condition-monitoring tasks, such as predicting equipment health, EDA supports the selection of meaningful features and clarifies how operational variables interact with failure modes.

**Figure 3:**
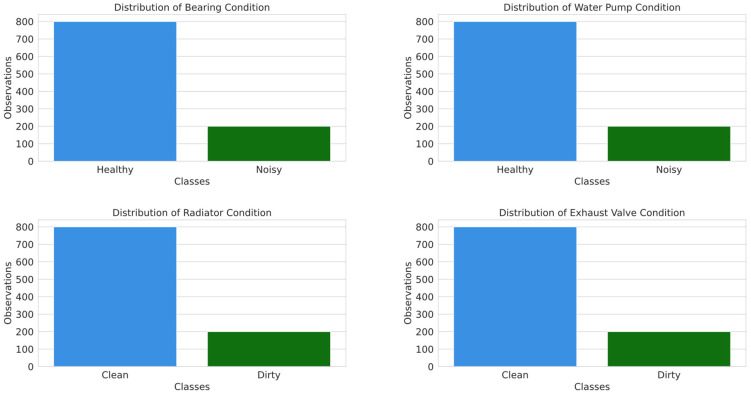
Overview of the distribution of the dependent variables that are provided in the dataset.

The dataset was collected from a water-cooled, two-piston, single-stage air compressor driven by an AC electric motor, capable of providing a maximum of 8 bars of compressed air [28]. The dataset contains 1000 observations, encompassing 24 features, from which 20 independent variables and 4 dependent variables can be included. The independent variables areas are as follows: motor RPM (rot/min), motor power (kW), motor torque (Nm), outlet pressure (bar), pressured air flow (L/min), compressor noise (dB), outlet temperature (°C), water pump outlet pressure (bar), water inlet and outlet temperatures (°C), water pump power (kW), water flow (L/min), oil pump power (kW), oil temperature (°C), and both ground acceleration and head acceleration (m/s^2^) (both measured in the x, y and z directions). The dependent variables represent the indicator classes of the four components for condition monitoring and fault detection, as shown in Figure 3. The bearing and water pump components were classified as either healthy or noisy, while the exhaust valve and radiator were classified as either clean or dirty.

In order to model the PdM-based solution, a subset of features was selected for each component, based on the domain knowledge of the system. For exhaust valve condition prediction, the most informative features are related to airflow dynamics, thermal behavior, and system load. Variables such as air flow and outlet pressure are directly impacted by a dirty or partially obstructed valve, which restricts airflow and alters the system pressure. Moreover, the rising temperature of the outlet air and oil tank temperatures indicate accumulating thermal stress due to inefficient exhaust discharge. As the compressor compensates for these inefficiencies, the motor power and torque increase, and the RPM may also be adjusted as a system-level response. In the context of bearing condition monitoring, the most relevant features are those that capture mechanical stress and vibration. Vibration sensors, such as head and ground accelerations along the x, y, and z axes, are critical for detecting the irregular motion and imbalance associated with bearing wear, misalignment, or friction. Another important aspect is that acoustic signals, such as air compressor noise, also reflect bearing deterioration, as noisy components often generate higher sound pressure levels. Operational load indicators, for instance, RPM, torque, and motor power, provide indirect insights into bearing health. For example, increased power or torque may indicate compensatory behavior in response to rising internal friction or degraded rotation. The selection of input features for water pump condition prediction is based on the physical principles of the governing pump operation. Specifically, variables such as water pump outlet pressure, water pump power, and water flow directly characterize the pump’s hydraulic and mechanical performance. Outlet pressure serves as a key indicator of flow resistance and internal efficiency, while power consumption reflects the mechanical load and potential degradation, due to factors such as friction, wear, or cavitation. Water flow is one of the most critical indicators, as a decline in flow typically signals blockages, impeller damage, or pumping capacity loss. Additionally, the selected feature set for radiator condition monitoring is based on the heat exchange efficiency’s thermodynamic and flow characteristics. For instance, the water flow is a critical indicator, as debris buildup or corrosion can impede flow through the radiator, resulting in reduced cooling performance. Moreover, water inlet and outlet temperatures are fundamental in assessing a radiator’s ability to dissipate thermal energy. A reduced temperature differential or persistently high outlet temperature may indicate fouling or clogging, which are characteristic of a dirty radiator.

In order to model the PdM-based solution, a subset of features was selected for each component based on the domain knowledge of the system. For exhaust valve condition prediction, the most informative features are related to airflow dynamics, thermal behavior, and system load. Variables such as air flow and outlet pressure are directly impacted by a dirty or partially obstructed valve, which restricts airflow and alters the system pressure. Moreover, the rising temperature of the outlet air and oil tank indicates accumulating thermal stress due to inefficient exhaust discharge. The motor power and torque increase as the compressor compensates for these inefficiencies, and the RPM may also be adjusted as a system-level response. In the context of bearing condition monitoring, the most relevant features are those that capture mechanical stress and vibration. Vibration sensors, such as head and ground accelerations along the x, y, and z axes, are critical for detecting the irregular motion and imbalance associated with bearing wear, misalignment, or friction. Another important aspect is that acoustic signals, such as air compressor noise, also reflect bearing deterioration, as noisy components often generate higher sound pressure levels. Operational load indicators, for instance, RPM, torque, and motor power, provide indirect insights into bearing health. For example, increased power or torque may indicate compensatory behavior in response to rising internal friction or degraded rotation. The selection of input features for water pump condition prediction is grounded in the physical principles that govern the pump operation. Specifically, variables such as water pump outlet pressure, water pump power, and water flow directly characterize the pump’s hydraulic and mechanical performance. Outlet pressure is a key indicator of flow resistance and internal efficiency, while power consumption reflects the mechanical load and potential degradation due to factors such as friction, wear, or cavitation. Water flow is one of the most critical indicators, as a decline in flow typically signals blockages, impeller damage, or pumping capacity loss. Additionally, the selected feature set for radiator condition monitoring is based on the heat exchange efficiency’s thermodynamic and flow characteristics. For instance, the water flow is a critical indicator because debris buildup or corrosion can impede flow through the radiator, resulting in reduced cooling performance. Moreover, water inlet and outlet temperatures are fundamental in assessing a radiator’s ability to dissipate thermal energy. A reduced temperature differential or persistently high outlet temperature may indicate fouling or clogging, which are characteristic of a dirty radiator.

**Figure 4:**
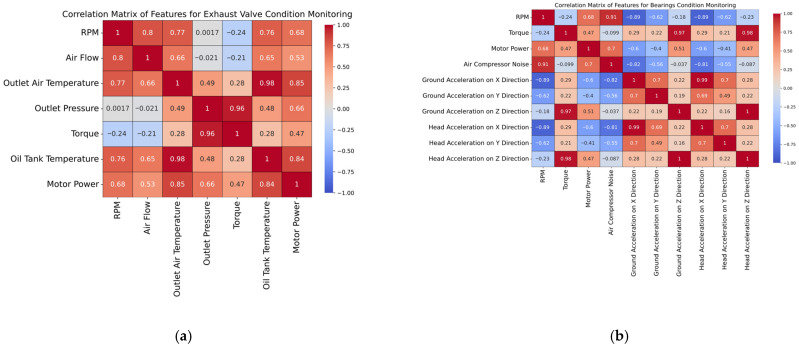
Overview of the correlation matrix of (**a**) exhaust valve and (**b**) bearing-related features.

**Figure 5:**
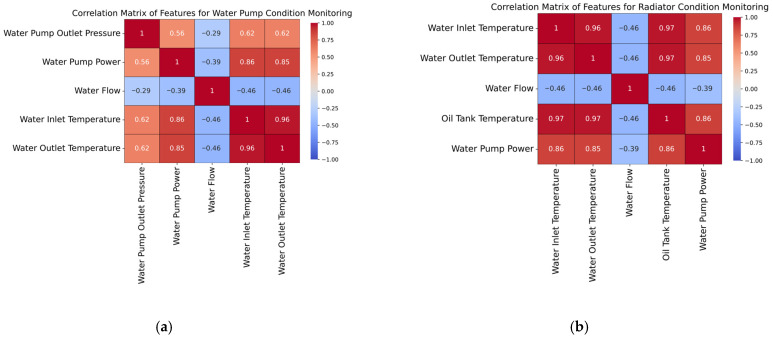
Overview of the correlation matrix of (**a**) water pump and (**b**) radiator—related features.

To gain a visual understanding of the correlation between the features characterizing both the exhaust valve and the bearings condition, two correlation matrices were constructed, as shown in Figure 4. Additionally, Figure 5 highlights the correlation matrices of the independent features used to predict the water pump and radiator conditions, respectively. In the case of the first correlation matrix (e.g., Figure 4a), the relationships are mainly driven by thermodynamic and mechanical interactions. For instance, outlet air and oil tank temperatures are highly correlated (e.g., 0.98), which is expected, since both reflect thermal stress in the system. The torque and outlet pressure also exhibit a strong correlation (e.g., 0.96), indicating that higher resistance leads to increased pressure and torque requirements. In fact, a dirty exhaust valve creates resistance. Therefore, the system compensates by increasing the pressure required to force air through, and the motor exerts more torque to maintain operation; both of these are measurable signs of potential exhaust valve degradation.

In the case of the second correlation matrix (i.e., Figure 4b), strong positive correlations can be observed among the ground and head acceleration signals (e.g., ground acceleration on X and head acceleration on X with a value of 0.99), suggesting that vibration patterns across different sensor placements and axes are closely linked. However, RPM shows a strong negative correlation with all acceleration features (i.e., up to −0.89), which may indicate that higher rotational speeds are associated with smoother operation and lower vibration, potentially reflecting healthy bearing behavior. In addition to acceleration-based features, several other operational metrics offer valuable insights into bearing condition. RPM and air compressor noise display a strong positive correlation (i.e., 0.91), indicating that the compressor noise increases proportionally with speed. Motor power shows strong positive correlations with both RPM (i.e., 0.68) and air compressor noise (i.e., 0.70), reinforcing its role as a central driver of system dynamics. The strongest correlations are observed between torque and ground acceleration in the Z direction (i.e., 0.97), as well as head acceleration in the same direction (i.e., 0.98), highlighting that load variations may manifest most noticeably in vertical vibrations.

Regarding the correlation matrix for the water pump condition (i.e., Figure 5a), water inlet and outlet temperatures are notably strongly correlated (0.96), which is expected in a thermal system where heat transfer is consistent. Water pump power also shows a high positive correlation with both water inlet (0.86) and water outlet temperatures (0.85), indicating that the increased thermal load corresponds to increased pump energy demand. Water pump outlet pressure displays a moderate correlation with temperature-related features (0.62), and power (0.56), suggesting its partial dependence on thermal and mechanical states. In contrast, water flow is negatively correlated with most features, implying that lower flow is associated with higher heat buildup, reflecting an incipient state of degradation. The correlation matrix for radiator condition monitoring (i.e., Figure 5b) reveals strong positive relationships among temperature-related features, namely water inlet, water outlet, and oil tank temperatures, as well as their shared correlation with water pump power. This indicates that thermal buildup within the system is closely tied to increased pump workload. Notably, water flow exhibits a consistent negative correlation with both temperature and power, suggesting that a reduced flow contributes to inefficient heat dissipation and increased system strain.

**Figure 6:**
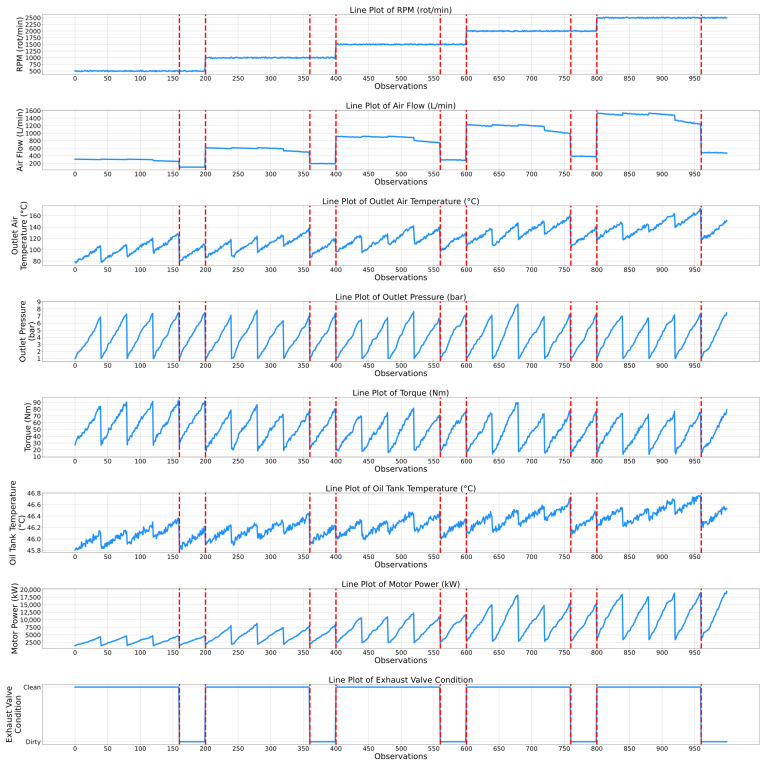
Temporal evolution of the exhaust valve condition, based on the selected features using domain knowledge. The blue solid lines represent measured values of each feature across the observation period, while the red dashed lines mark maintenance events.

**Figure 7:**
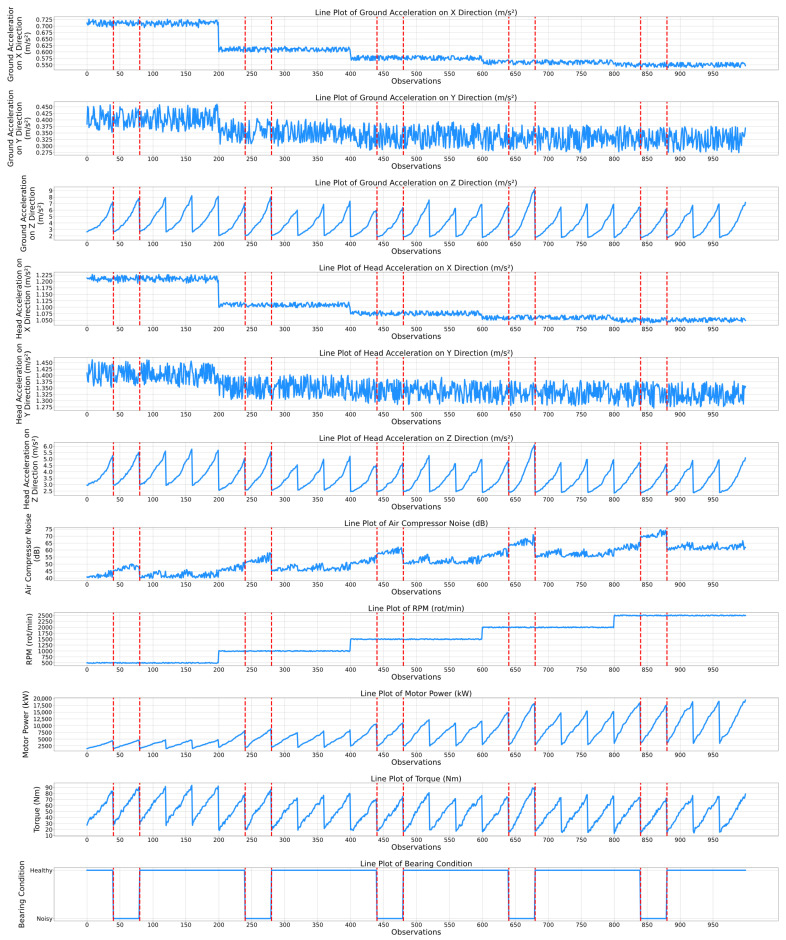
Temporal evolution of the bearing component condition, based on the selected features using domain knowledge. The blue solid lines represent measured values of each feature across the observation period, while the red dashed lines mark maintenance events.

**Figure 8:**
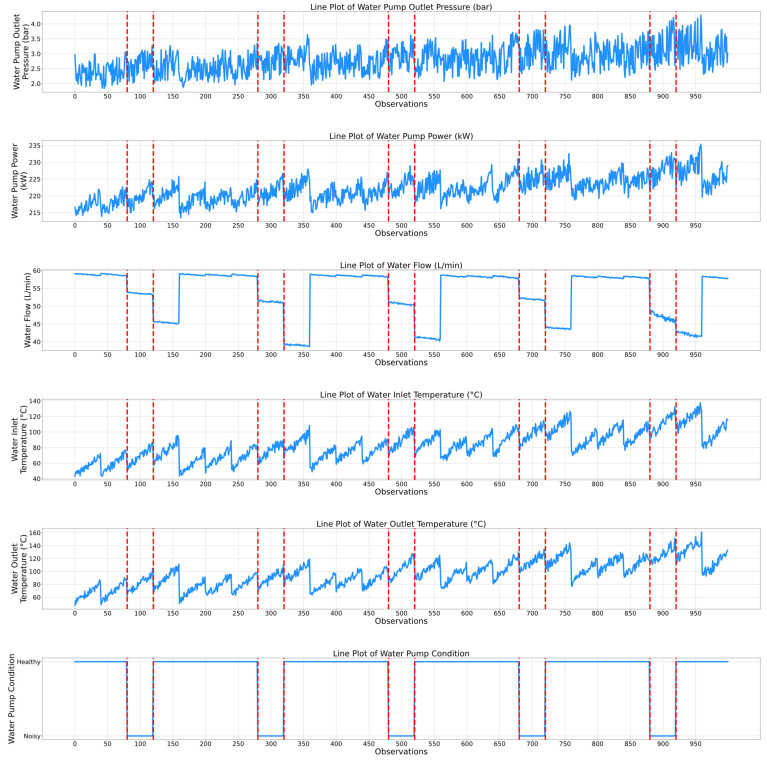
Temporal evolution of the water pump condition, based on the selected features using domain knowledge. The blue solid lines represent measured values of each feature across the observation period, while the red dashed lines mark maintenance events. Temporal plot highlighting the evolution of the bearings component’s condition, based on the selected features.

**Figure 9:**
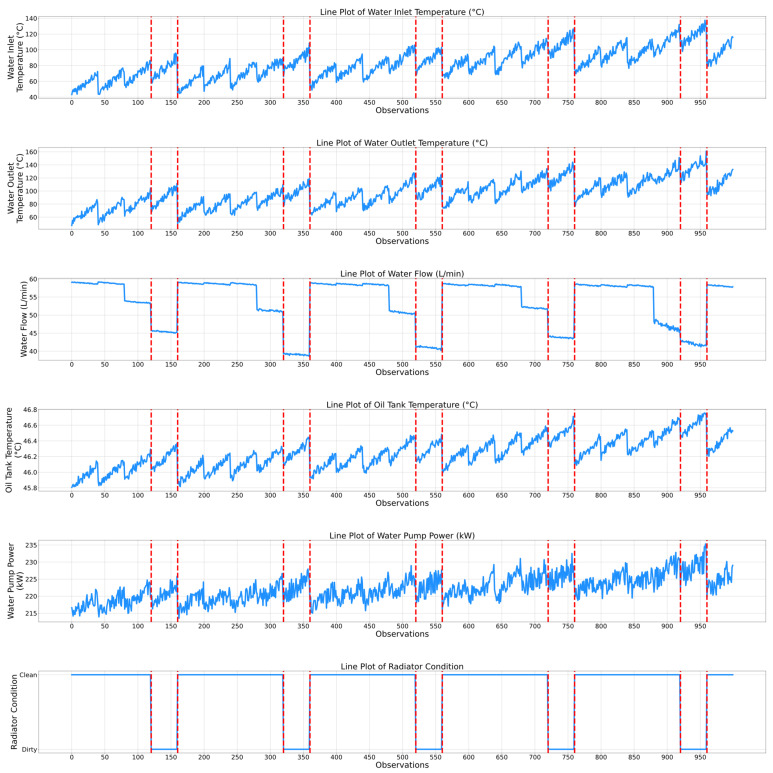
Temporal evolution of the radiator condition, based on the selected features using domain knowledge. The blue solid lines represent measured values of each feature across the observation period, while the red dashed lines mark maintenance events. Temporal plot highlighting the evolution of the water pump condition, based on the selected features. Temporal plot highlighting the evolution of the bearings component’s condition, based on the selected features.

While the previously presented correlation matrices highlight a static relationship between the features of the air compressor components, temporal evaluation plots present a complementing dynamic viewpoint by showing how the features and the associated condition indicators of the air compressor components change over time. Thus, the maintenance personnel can observe not only which features are related, but also how their dynamics correspond to specific events, transitions, or anomalies in the air compressor system. Therefore, Figure 6 and Figure 7 show the temporal plots for the exhaust valve and bearings components, while Figure 8 and Figure 9 highlight the dynamic behavior for the water pump and radiator, respectively, as a function of the selected features.

In Figure 6, it can be observed that the “Air Flow” and RMP features show clear stepwise variations throughout the timeframe. Notably, the changes in the features’ values correspond with all the variations in the exhaust valve condition, indicating a strong operational correlation. For instance, within the first 200 observations, when the value of the air flow decreases from 300 L/min to about 100 L/min, the condition of the exhaust valve changes from clean to dirty. This trend can be further observed as the number of observations increases (i.e., and implicitly, the degradation level of the exhaust valve also increases). In fact, the most clearly observed correlation between the air flow and the condition of the exhaust is within the last functional cycle (i.e., between 800 and 1000 observations), when the condition clearly changes from clean to dirty as the air flow value decreases from 1200 L/min to 500 L/min. Similarly, the RPM feature tends to align with the condition of the exhaust valve as well. For instance, it can be observed that when the exhaust valve makes a transition from the dirty state to the clean state, the RPM undergoes an upward adjustment in value (i.e., from 1000 to 1500 at the 400th observation, or from 2000 to 2500 at the 800th observation), indicating that maintenance restores the exhaust valve’s capacity, allowing for higher operational speeds and improved performance. Additionally, the “Outlet Air Temperature” feature is also closely related to the condition of the exhaust valve. For instance, in most functioning cycles (i.e., until the 200th, 400th, 600th or 800th observations) the peak-to-peak difference when the exhaust valve condition changes from clean to dirty is 50 °C, while the peak-to-peak difference is only 20 °C, indicating that the exhaust valve experiences much greater temperature fluctuations when it is clean compared to when it is dirty. Therefore, a clean exhaust valve allows for more temperature variance, while a dirty valve constrains this range, suggesting less system responsiveness or efficiency.

Figure 7 highlights the temporal evolution of the bearings component as a function of ten features. It can be clearly observed that one of the most correlated features with its condition (i.e., healthy or noisy) is the “Air Compressor Noise” feature. In fact, a clear ascending trend can be observed as the number of observations increases. Notably, in every functioning cycle, the value of the noise in the air compressor’s bearings is significantly higher when the system is diagnosed as noisy. In fact, from the 40th observation until the 80th observation (i.e., noisy condition), the peak noise value is approximately 50 dB, compared to 40–45 dB recorded within the first 40 observations. Additionally, the difference in noise values between different conditions tends to increase as the number of observations increases. For example, between the 640th and 680th observations, the recorded noise value varied between 65 and 70 dB. Based on the previously presented analysis, the “Air Compressor Noise” feature can be considered a key degradation indicator of the bearings component. Another relevant degradation indicator is the “Motor Power” feature. Within the first 640 observations, no outstanding variations can be observed when the state changes between healthy and noisy. However, between the 640th and 680th observations, the peak value of the motor power is 17,500 kW, which is slightly higher (i.e., by 500 kW) than the values recorded in the previous observations.

Figure 8 presents the temporal evolution of the water pump component within the air compressor. As expected, one of the key degradation indicators of this component is the “Water Flow” feature, since it is highly correlated with the corresponding condition indicator. Notably, at the moments when the water pump condition changes from healthy to noisy, the water flow clearly decreases. Additionally, in every operational cycle, the water flow rate remains relatively constant. For instance, the water flow value within the first 80 observations is 58–59 L/min, corresponding to the healthy condition. Next, when the water pump becomes noisy (i.e., between the 80th and 120th observations), the water flow value decreases from 58 to 59 L/min to 54–55 L/min. Moreover, it can be observed that the lowest water flow rate values that were recorded (i.e., 45 L/min between the 120th and 160th observations or 41 L/min between the 520th and 560th observations) correspond to the healthy state of the water pump, which may not properly match the expected physical behavior. However, this behavior only occurs for approximately 40 observations per functioning cycle, after which the water flow rate resets at the optimal value, which is approximately 58–60 L/min. Another feature that has been utilized to predict the state of the water pump is represented by the water inlet/outlet temperature. Both the water inlet and outlet temperature features exhibit higher values when the water pump is in the noisy state compared to the recorded values corresponding to the healthy state. For instance, the peak water inlet temperature that was recorded between the 480th and 530th observations was approximately 130 °C, which is 20 °C higher than the values that correspond to the healthy state from the corresponding functioning cycle.

Figure 9 presents the temporal evolution of the radiator component of the air compressor. Similarly to the water pump case, the water flow feature represents a key degradation indicator. Unlike the previous case, when the condition of the water pump is classified as noisy, the water flow rate is at a minimum in every functioning cycle. For instance, between the 160th and 280th observations, the water flow rate is relatively constant, then, as the radiator approaches the dirty state, it drops from 58 to 59 L/min to approximately 51 L/min. When the radiator reaches the dirty state (i.e., between the 320th and 360th observations) the water flow rate value drops even further, to approximately 40 L/min. Therefore, this feature clearly indicates the radiator degradation state. Additionally, the oil tank temperature also consistently highlights the degradation level of the radiator. In fact, in almost every functioning cycle, the peak temperature values of the oil tank are higher when the condition of the radiator is dirty compared to the clean state. For example, between the 560th and 720th observations (i.e., clean radiator), the mean recorded temperature is approximately 46.3 °C, compared to 46.7 °C, which is the peak temperature recorded between the 720th and 760th observations (i.e., dirty radiator). The other selected features, such as the water outlet temperature or the water pump power, seem to have the same behavior as the oil tank temperature (i.e., higher value when the condition of the radiator is classified as dirty and lower values when the condition of the radiator is classified as clean).

Rather than solely relying on domain knowledge for feature selection, the chi-square test was also used to compare these results with the ones obtained by the SHAP feature importance diagrams. The chi-square test represents a hypothesis test that can be used to determine whether features from the dataset are dependent/independent with the target variables. Formally, the chi-square is computed according to Equation (1).

(1) $$\chi^{2}=\sum_{i=1}^{n}\frac{{\left(O_{i}-E_{i}\right)}^{2}}{E_{i}}$$

where $O_{i},(i=1,2,\ldots ,n)$ represents the set of observed values and $E_{i},(i=1,2,\ldots ,n)$ represent the corresponding set of expected values.

### 2.2. The Architecture of the DL-Based Model

**Figure 10:**
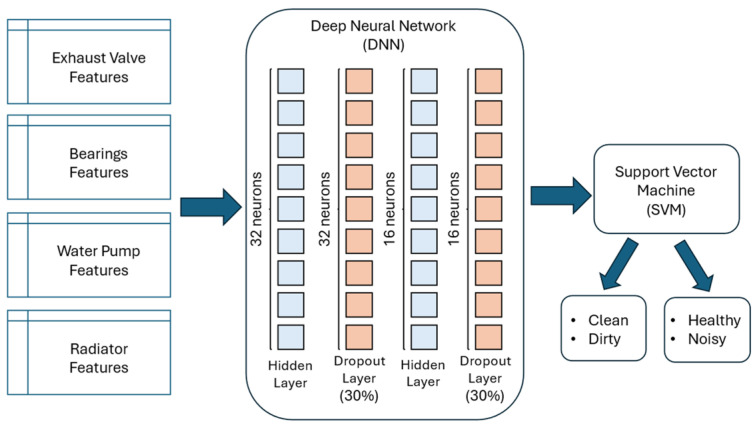
Overview of the hybrid DL-based model for air compressor components condition monitoring and fault detection.

Figure 10 presents the architecture of the hybrid DL-based model developed for condition monitoring and fault detection of the exhaust valve and bearings.

The architecture of the hybrid DL-based model encompasses a deep neural network (DNN) for extracting the related features, followed by an SVM module that is responsible for the final fault detection classification task. The DNN is composed of two layers of fully connected neurons (i.e., 32 neurons in the first hidden layer and 16 neurons in the second hidden layer). After each hidden layer, a dropout layer was introduced with a dropout rate of 30%, in order to mitigate overfitting. Since the number of observations is limited (i.e., 1000 observations), introducing a dropout layer after each hidden layer enhances the overall robustness of the hybrid model. Additionally, the radial basis function kernel was used in conjunction with the SVM algorithm, since it can efficiently map the features into a higher-dimensional space where a nonlinear decision boundary becomes linearly separable. Formally, the architecture of the hybrid DL-based model is presented in Equation (2).

(2) $$\hat{y}=sign\left(w^{⊺}\sigma_{2}\left({Drop}_{0.3}\left(W_{2}\sigma_{1}\left({Drop}_{0.3}\left(W_{1}x+b_{1}\right)\right)+b_{2}\right)\right)+b\right)$$

where *x* represents the input feature vector of each air compressor component individually, $W_{i}$ and $b_{i}$ represent the weights and biases of the first and second hidden layers, $\sigma_{i}$, i=1,2 represents the activation function, ${Drop}_{0.3}$ represents the dropout layer and $\hat{y}$ is the predicted condition of each component of the air compressor.

From the distribution of the target variables, presented in Figure 3, it can be clearly observed that the data are highly imbalanced, with 80% of the observations classified as clean/healthy and only 20% as dirty/noisy. Therefore, we employed the adaptive synthetic oversampling (ADASYN) technique, which adaptively focuses on the instances that are near the decision boundary or surrounded by majority class observations, generating more synthetic data where they are most needed and improving the classifier’s reliability when utilized in conjunction with SVM [29]. Moreover, the ADASYN technique was only applied to the training set in order to avoid introducing bias into the model.

To validate the robustness of the DL-based architecture, several performance metrics were used: accuracy (Equation (3)), precision (Equation (4)), recall (Equation (5)) and F1-score (Equation (6)).

(3) $$Accuracy=\frac{TP+TN}{TP+TN+FP+FN}$$

(4) $$Precision=\frac{TP}{TP+FP}$$

(5) $$Recall=\frac{TP}{TP+FN}$$

(6) $$F1\text{-}Score=\frac{2\cdot Precision\cdot Recall}{Precision+Recall}$$

Regarding the hardware equipment used during both the training and inference phases, the NVIDIA T4 GPU with 8 GB of RAM, a Raspberry Pi 4 Model B board with 4 GB of RAM and an NVIDIA Jetson Nano development kit with 4 GB of RAM LPDDR4 memory were utilized. Additionally, the software was developed using the TensorFlow framework, version 2.12.0, as well as the Scikit-learn version 1.2.2 library. The hyperparameters values used for training the DNN feature extractor are presented in Table 2. Since condition monitoring for both the exhaust valve and bearings can be formulated as a binary classification task, the loss function used is represented by the binary cross-entropy function. Since the number of observations from the dataset is relatively reduced, the five-fold stratified cross-validation technique was employed, rather than a simple train/test split. In contrast to a conventional train/test split, which can introduce random imbalances in class distributions and consequently yield unreliable performance estimates, stratified k-fold cross-validation systematically preserves the original class proportions within each fold. Stratification enhances the consistency and fairness of the evaluation process, resulting in more stable metrics across folds and improved reliability when assessing model performance.

### 2.3. Explainable AI Methods

XAI represents a key factor in increasing the transparency, accountability, and general adoption of ML- and DL-based models. One of the most popular XAI methods which is widely adopted is represented by the SHAP diagrams [30], which can generate consistent, locally accurate explanations by attributing each feature’s contribution to a specific prediction, based on cooperative game theory. Consider a DL-based model denoted as f and a set of input features $x\in R^{n}$. Then, the SHAP explanation expresses the model output as an additive combination of feature contributions as shown in Equation (7).

(7) $$f\left(x\right)=\sum_{i=1}^{n}\phi_{i}+\phi_{0},\;\;\phi_{i}=E[f(x)]$$

In the above equation, $\phi_{0}$ represents the expected model output and each $\phi_{i}$ represents the marginal contribution of each feature to the final prediction. Additionally, the value of $\phi_{i}$ is computed by averaging its impact over all possible subsets *S* of features N={1, 2,…, n}, excluding the ith feature, weighted by the size of the subset as shown in Equation (8). Therefore, this equation ensures that each feature’s contribution is calculated fairly, accounting for all possible feature orderings. Additionally, the cardinal of N varies depending on the component (i.e., Card(N) = 7 in the case of the exhaust valve, Card(N) = 10 in the case of the bearings and Card(N) = 5 in the case of both the water pump and radiator).

(8) $$\phi_{i}=\sum_{S\subseteq N\backslash \{i\}}\frac{\left|S\right|!\left(n-\left|S\right|-1\right)!}{n!}\left[f_{S\;\cup \;\left\{i\right\}}(x_{S\;\cup \;\left\{i\right\}}-f_{S}(x_{S}))\right]$$

In this paper, two visualization techniques were utilized to highlight the SHAP values: bar plots and beeswarm plots. The SHAP bar chart provides a global view of feature importance by displaying the average absolute SHAP value for each feature across the entire dataset, measured along the X axis. This plot answers the question: “Which features have the greatest overall impact on model output?”. Therefore, for the *i*th considered feature for predicting the condition of the air compressor components, the global importance is defined in Equation (9):

(9) $${IMP}_{i}=\;\frac{1}{m}\sum_{j=1}^{m}\left|\phi_{i}^{\left(j\right)}\right|$$

where m represents the number of instances in the dataset and $\phi_{i}^{\left(j\right)}$ represents the SHAP value of the *i*th instance and *j*th feature. On the other hand, the SHAP beeswarm plot offers a more granular, instance-level view by visualizing the full value distribution for each feature. It highlights not only the magnitude of each feature’s effect but also the direction (i.e., either positive or negative) and the relationship between raw feature values and their corresponding contributions to the prediction. The X-axis shows the direction and magnitude of this contribution, indicating how strongly and in which direction feature *i* influences the model’s prediction. The color of each point reflects the raw feature value $x_{i}^{\left(j\right)}$, with red indicating high values and blue representing low values. The density spread along the X-axis illustrates the distribution of SHAP values, where wider regions signify more frequent occurrences of similar $\phi_{i}^{\left(j\right)}\;$values across instances. This visualization provides a detailed summary of both the global importance and instance-level behavior of each feature. The pseudocode that briefly shows how SHAP diagrams have been generated is presented in Algorithm 1 below.
**Algorithm 1**: SHAP Visualization
$\mathrm{Require}:\;D_{train}=\left\{\left(X_{1},Y_{1}\right),\left(X_{2},Y_{2}\right),\ldots ,\left(X_{N},Y_{N}\right)\right\}$, $D_{test}=\left\{\left(X_{1}^{\prime },Y_{1}^{\prime }\right),\left(X_{2}^{\prime },Y_{2}^{\prime }\right),\ldots ,\left(X_{N}^{\prime },Y_{N}^{\prime }\right)\right\}$, number of folds K = 5 1:**for each** fold k = 1, 2, …, K **do**:2:
$\mathrm{Train}\;f_{DNN-SVM}$$\;\mathrm{on}\;D_{train}^{k}$3:
$\mathrm{Fit}\;\mathrm{SHAP}\;\mathrm{DeepExplainer}(f_{DNN-SVM},\;D_{train}^{k}$)4:
$\mathrm{Compute}\;\mathrm{the}\;\mathrm{SHAP}\;\mathrm{values}\;\phi_{i}$$\;\mathrm{according}\;\mathrm{to}\;\mathrm{Equation}\;(7)\;\mathrm{on}\;D_{test}$5:**end for each**6:$\mathrm{Aggregate}\;\mathrm{SHAP}\;\mathrm{values}\;\phi_{i}$ across folds7:$\mathrm{Compute}\;\mathrm{the}\;\mathrm{global}\;\mathrm{importance}\;\mathrm{of}\;\mathrm{SHAP}\;\mathrm{values}\;\mathrm{according}\;\mathrm{to}\;\mathrm{Equation}\;(8)\;\mathrm{on}\;D_{test}$8:Visualize SHAP bar and beeswarm plots

LIME [31] provides a complementary, local view of feature importance by approximating the model’s decision boundary in a single instance. In the context of the air compressor, LIME was employed to visually illustrate the decision logic of the fault-diagnosis model on a per-instance basis. For each selected compressor state (i.e., the exhaust valve’s “Clean” condition or the water pump’s “Healthy” classification), LIME generates slight perturbations of the relevant sensor features (e.g., air flow, RPM, water flow, etc.) in the vicinity of the observation and fits a sparse linear surrogate to locally approximate the model’s decision boundary. The resulting bar charts rank and quantify each feature’s contribution, with green bars indicating increased target class probability and red bars indicating decreased probability. Formally, LIME addresses the optimization problem shown in Equation (10).

(10) $$\xi (x)=\underset{g\in G}{\mathrm{arg min}}\left[L\left(f,\;g,\;\pi_{x}\right)+\Omega (g)\right]$$

where x represents the instance to be explained, f represents the hybrid DL-based model, G is the family of simple interpretable models (i.e., sparse linear models), $L\left(f,\;g,\;\pi_{x}\right)$ represents the local fidelity term (Equation (11)) and Ω(g) is a complexity penalty on the surrogate model g. Therefore, model g remains simple and interpretable. Additionally, L quantifies the local fidelity by measuring the weighted squared error between the black-box predictions f(z) and the surrogate outputs g(z) over perturbed samples z near the input feature x.

(11) $$L\left(f,\;g,\;\pi_{x}\right)=\sum_{z\in Ζ}\pi_{x}(z){\left[f\left(z\right)-g(z)\right]}^{2}$$

Therefore, LIME aims to find, for a specific input feature x, a simple model g that optimally trades off between two objectives: local fidelity, ensuring *g* approximates the black-box predictor accurately in the vicinity of x (i.e., quantified by the $L\left(f,\;g,\;\pi_{x}\right)$) and model simplicity, enforcing interpretability through a complexity penalty Ω(g). The pseudocode that briefly shows how LIME diagrams have been generated is presented in Algorithm 2 below.
**Algorithm 2**: LIME Visualization
$\mathrm{Require}:\;{F=\{f}_{1},\;f_{2},\;\ldots ,f_{N}\}$ features of each air compressor component 1:Select top N features based on SHAP importance2:$\mathrm{Fit}\;\varepsilon_{LIME}=LimeTabularExplainer$$\;\mathrm{on}\;D_{train}[:,F]$3:$\mathrm{Identify}\;\mathrm{the}\;\mathrm{set}\;S\;\mathrm{of}\;\mathrm{high}\text{-}\mathrm{confidence}\;\mathrm{predictions}\;x_{i}$4$\mathrm{for}\;\mathrm{each}\;x_{i}\;\in S$ **do:**5:
$\mathrm{Determine}\;\mathrm{local}\;\mathrm{explanations}\;\omega_{i}=\varepsilon_{LIME}.explain\_instance(x_{i},\;f_{DNN-SVM})$6:
$\mathrm{Visualize}\;\mathrm{local}\;\mathrm{explanations}\;\omega_{i}$7:**end for each**

The third XAI method that is employed in visualizing the impact of the features over the DL-based model is PDP [32]. PDP is a global, model-agnostic tool that visualizes the averaged effect of one input or two features to the predicted outcome of the DNN. A partial dependence function for a feature j is formally defined as the expected model prediction when we fix j to a value z, and average out all other features as shown in Equation (12).

(12) $${PD}_{j}=E_{X_{\backslash j}}\left[f\left(X_{j}=z,\;X_{\backslash j}\right)\right]=\int f(z,\;X_{\backslash j})dP_{X_{\backslash j}}(X_{\backslash j})$$

where $X_{\backslash j}$ denotes the vector of all the features characterizing each component of the air compressor except the jth feature, f represents the DNN model and $P_{X_{\backslash j}}$ denotes the joint distribution. Additionally, when a PDP curve is strictly monotonic (i.e., either increasing or decreasing), it reveals a consistent positive or negative association between that feature and the target class. In contrast, a U or bell-shaped PDP curve identifies the presence of an optimal operating window, showing that intermediate values of the feature maximize predictive confidence while extremes on either side reduce it. Finally, a largely flat PDP curve indicates that variations in that feature have minimal effect on the model’s output, suggesting that it contributes little to the decision boundary. The pseudocode that briefly presents how PDP diagrams have been generated is presented in Algorithm 3 below.
**Algorithm 3**: PDP Explanations Visualization
$\mathrm{Require}:\;\mathrm{Trained}\;f_{DNN-SVM}$$,\;D_{test}$$,\;\mathrm{most}\;\mathrm{influential}\;\mathrm{feature}\;\mathrm{determined}\;\mathrm{by}\;\mathrm{SHAP}\;j^{*}$, number of grid points G1:$\mathrm{Construct}\;a\;\mathrm{grid}\;\mathrm{of}\;\mathrm{values}\;G=\{v_{1},\;v_{2},\;\ldots ,v_{n}\}$$\;\mathrm{spanning}\;\mathrm{the}\;\mathrm{range}\;\mathrm{of}\;j^{*}$$\;\mathrm{in}\;D_{test}$2:$\mathrm{for}\;\mathrm{each}\;v_{i}\;\in G$ **do:**3:
$\mathrm{Construct}\;a\;\mathrm{copy}\;D_{test}^{\prime }$4:
$\mathrm{Set}\;\mathrm{the}\;j^{*th}$$\;\mathrm{featureof}\;\mathrm{all}\;\mathrm{samples}\;\mathrm{in}\;D_{test}^{\prime }$$\;\mathrm{to}\;v_{i}$$D_{test}^{\prime }\left[:,j^{*}\right]=v_{i}$5:
$\mathrm{Obtain}\;\mathrm{predicted}\;\mathrm{values}\;\hat{y}=f_{DNN-SVM}(D_{test}^{\prime })$6:
$PDP\left(v_{i}\right)=\frac{1}{n}\sum_{i=1}^{n}{\hat{y}}_{i}$7:**end for each**8:$\mathrm{Plot}(\left\{v_{1},\;v_{2},\;\ldots ,v_{n}\right\},\;{PDP(v}_{1},\;v_{2},\;\ldots ,v_{n})$

## 3. Results and Discussions

This section presents the experimental results obtained after training and validating the hybrid DL-based model, DNN model, and SVM architecture on NVIDIA T4 GPU, Raspberry Pi 4 Model B, and NVIDIA Jetson Nano. Additionally, the SHAP, PDP, LIME and ROC diagrams obtained after model training and validation are presented in this section. Moreover, a comparison between the pure DNN and the standalone SVM model is highlighted in terms of both performance and interpretability.

### 3.1. Results Obtained Using Hybrid Architecture

The performance results, in terms of accuracy, precision, recall, and F1-score, which were obtained after training and by validating the hybrid model on NVIDIA T4 GPU, Raspberry Pi 4 Model B, and NVIDIA Jetson Nano GPU, are presented in Table 3, Table 4 and Table 5. The model demonstrated strong and consistent performance across all platforms, with only marginal differences across the evaluated metrics. For instance, in the case of the exhaust valve component, the F1-score was 98.3% on the NVIDIA T4 GPU, improved to 98.7% on the Raspberry Pi 4, and remained consistent at 98.7% on the Jetson Nano. Similarly, for the bearing component, the F1-score increased slightly from 98.6% on the GPU to 98.9% on the Raspberry Pi, and was comparable at 98.9% on the Jetson Nano. Across all components, precision and recall values remained above 98%, indicating the model’s robustness and ability to maintain high classification accuracy with minimal false positives and false negatives. These results highlight the model’s efficiency and generalization capability, making it well-suited for edge computing scenarios, including deployment on resource-constrained devices, such as the Raspberry Pi.

Additionally, Table 6, Table 7 and Table 8 present the device-level runtime performance of the hybrid DL-based model across five folds for the entire set of air compressor components (i.e., exhaust valve, bearings, water pump, and radiator) on all the previously mentioned devices. As expected, the training and inference times on the Raspberry Pi are higher, due to its limited computational capabilities. The average training time per epoch on the GPU ranged from approximately 136 to 154 ms, whereas on the Raspberry Pi, it ranged between 641 and 799 ms, which is approximately 4.5 to 5 times slower. Similarly, the average inference time per epoch on the GPU was under 70 ms for both conditions, while on the Raspberry Pi, it consistently remained below 240 ms. The NVIDIA Jetson Nano GPU demonstrates a significant improvement in inference efficiency over the Raspberry Pi 4, achieving lower inference times across all components. For instance, the average inference time per epoch for the exhaust valve condition is approximately 13.9 ms on Jetson Nano, compared to 227.2 ms on Raspberry Pi and 62.8 ms on the NVIDIA T4 GPU. This pattern is consistent across all tasks, with Jetson Nano achieving between 4 and 17 times faster inference times than the Raspberry Pi and outperforming the T4 in latency, despite having lower computational power. In terms of training time, the Jetson Nano GPU performs faster than the Raspberry Pi, but remains significantly slower than the NVIDIA T4 GPU. For example, the training times per epoch for the radiator condition average around 807 ms on Jetson Nano, compared to 647 ms on Raspberry Pi, and only 126 ms on the T4 GPU. This indicates that while the Nano is efficient for real-time or low-latency inference, it is less suited for fast model training. Given the nature of predictive maintenance, where sub-second responses are typically sufficient, these inference times are well-suited for near-real-time diagnostics. In terms of inference latency, Jetson Nano outperforms Nividia T4 due to several reasons. Firstly, Jetson is optimized for light-weight models, such as a two-layer DNN or SVM, due to the reduced kernel launch overhead compared to NVIDIA T4. Secondly, the CPU and GPU memory from the NVIDIA T4 are separated, whereas the Jetson Nano provides shared CPU and GPU memory. Therefore, in the case of both lightweight models and small tensors in terms of shape, the communication on a unified memory architecture like the one Jetson Nano provides is reflected in reduced latency compared to the distributed one provided by NVIDIA T4. This highlights the model’s practical deployability on low-power edge devices, enabling cost-effective and scalable monitoring solutions in industrial environments.

Table 9 highlights the memory usage, energy consumption, and CO_2_ emissions recorded on each hybrid model device. Regarding the recorded memory usage during the training phase, NVIDIA T4 exhibited the highest memory usage across the entire set of components, with over 2 GB of utilized memory, followed by NVIDIA Jetson Nano and Raspberry Pi model B. Both the energy consumption across each device and the quantity of carbon dioxide emissions have been determined using the CodeCarbon library [33]. The carbon dioxide emissions have been determined as the product between the carbon intensity of the electricity utilized during model training and the energy consumed by each device. As expected, the higher the energy consumption of a particular device, the higher the carbon dioxide emissions are exposed. However, given the reduced size of the dataset, both the energy consumption and carbon dioxide emissions are relatively reduced.

**Figure 11:**
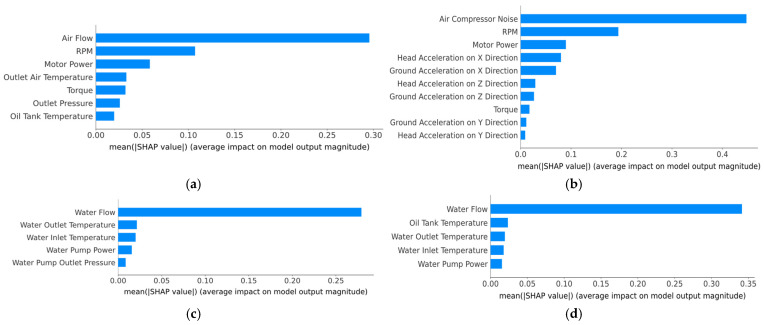
Overview of the bar plot SHAP diagrams of the hybrid model highlighting the feature importance using domain knowledge for (**a**) exhaust valve, (**b**) bearing, (**c**) water pump, and (**d**) radiator air compressor components.

The SHAP diagrams, which highlight the importance and contribution of each feature for all components within the air compressor of the hybrid model, are presented in Figure 11. For the exhaust valve condition prediction (i.e., Figure 11a), “Air Flow” exhibits the highest average contribution to the output of the model, followed by the “RPM” and “Motor Power”, indicating that the model predominantly relies on real-time air and mechanical flow dynamics to discriminate between clean and dirty valve states. For bearing condition prediction (i.e., Figure 11b), the bar plot shows that the “Air Compressor Noise” is by far the most influential feature in the inference phase, followed by the RPM and “Motor Power”. In contrast, the vibration and acceleration features, while relevant in this case, had relatively low impact on the model prediction, suggesting that the noise and power characteristics offered more reliable indicators of bearing health for this dataset. Regarding the water pump (Figure 11c) and radiator (Figure 11d) condition predictions, it can be observed that the “Water Flow” is by far the most influential feature for both components. The rest of the features (i.e., water inlet/outlet temperature, water pump power or oil tank temperature), while relevant, have a smaller influence on the decision-making process of the model.

**Figure 12:**
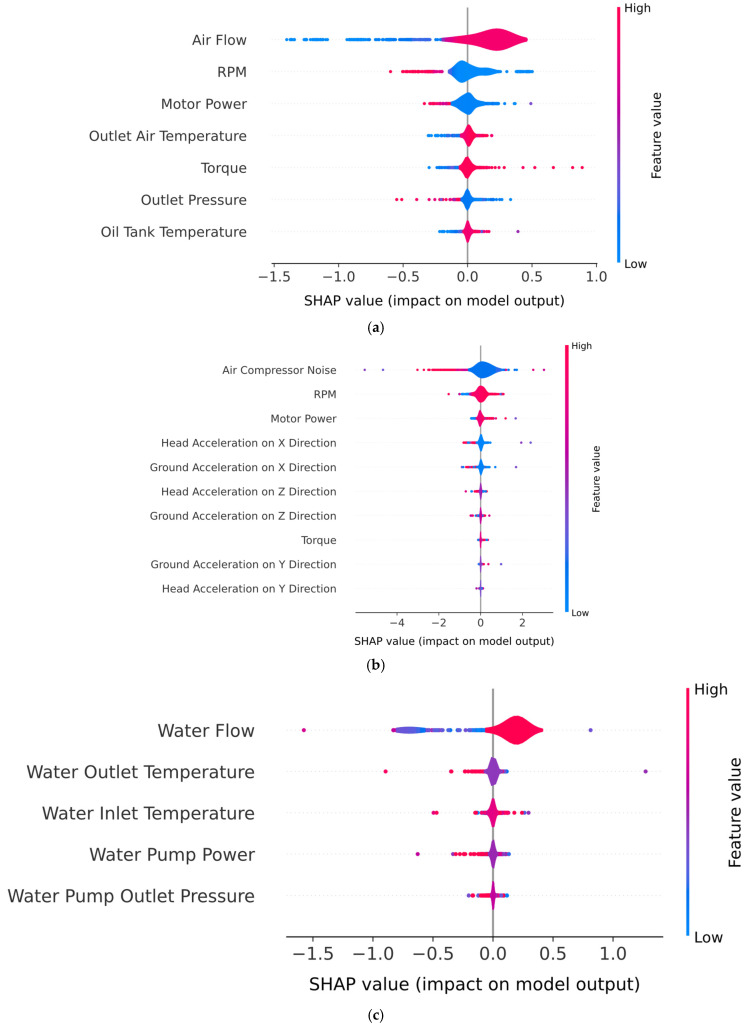

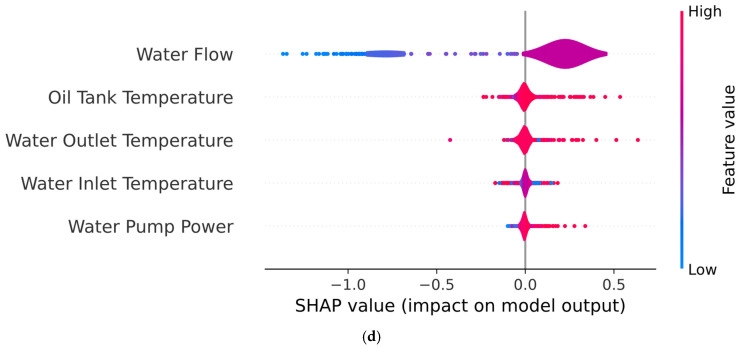
Overview of the beeswarm SHAP diagrams obtained by the hybrid model, highlighting the contribution of the selected features using domain knowledge for (**a**) exhaust valve, (**b**) bearing, (**c**) water pump, and (**d**) radiator air compressor components.

Figure 12 presents the beeswarm plots obtained by the hybrid model for all air compressor components. For the exhaust valve (Figure 12a), the beeswarm plot captures both the magnitude and direction of feature influence on individual predictions for the “Clean” class. Therefore, higher air flow values are consistently associated with positive SHAP values, indicating that increased air flow directly contributes to the model’s confidence in predicting a clean valve condition. Additionally, low air flow values are associated with negative SHAP values, which increases the probability of a dirty valve condition. A similar trend was observed for both RPM and motor power features, where lower values were associated with clean valve conditions, while higher values tended to indicate a dirty valve. In the case of the beeswarm plot for the bearings component (Figure 12b), the features with positive SHAP values push the prediction toward the “Healthy” class, while negative features shift the model toward predicting a noisy bearing. Therefore, low noise levels are mostly associated with positive SHAP values, suggesting that reduced mechanical noise increases the model’s confidence in predicting a healthy bearing state. Moreover, RPM and motor power show that the SHAP distributions are mostly centered at zero, with a slight skew toward negative values. Higher values, though typically neutral, are observed to modestly contribute to the model predicting a noisy bearing, reflecting increased system stress under degraded operating conditions. The SHAP Beeswarm plot for water pump condition monitoring (i.e., Figure 12c) reveals that “Water Flow” is the most influential feature, with higher flow values strongly associated with the prediction of a “Healthy” condition and lower values pushing the prediction toward “Noisy”. This aligns with the physical expectation that degraded pump performance often leads to reduced flow. “Water Outlet Temperature” also shows moderate discriminative power, with elevated temperatures slightly skewing the model’s output toward the “Noisy” class, indicating thermal stress or inefficiency. While “Water Inlet Temperature” and “Water Pump Power” contribute marginally to model decisions, “Water Pump Outlet Pressure” has limited predictive value. Regarding the SHAP diagrams for radiator condition monitoring (i.e., Figure 12d), the most influential feature is “Water Flow”, where high values consistently produce positive SHAP values, strongly pushing the model toward predicting a “Clean” condition. However, lower “Water Flow” values shift the model’s output away from the “Clean” condition, therefore favoring the “Dirty” class. Additionally, thermal features such as “Oil Tank Temperature”, “Water Outlet Temperature”, and “Water Inlet Temperature” also contribute to the prediction, albeit to a lesser extent. High temperature readings in these features are mostly centered around neutral or slightly positive SHAP values for high feature values, indicating that elevated system temperatures reduce the confidence of the model in classifying the radiator condition as “Clean”.

**Figure 13:**
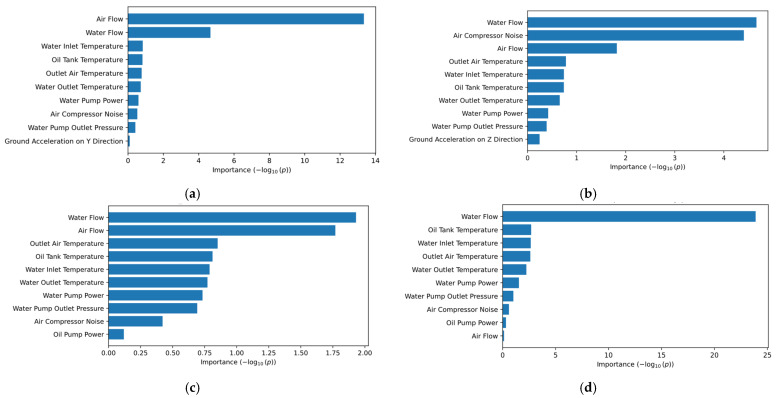
Overview of the bar plot SHAP diagrams highlighting the feature importance of the selected features using the chi-sqare test for (**a**) exhaust valve, (**b**) bearing, (**c**) water pump, and (**d**) radiator air compressor components.

Figure 13 highlights the top ten feature importance rankings obtained after applying the chi-square test. It can be observed that across most of the air compressor components (i.e., exhaust valve (Figure 13a), water pump (Figure 13c), and radiator (Figure 13d)), the most influential features determined by the chi-square test correspond to the one determined by SHAP. Additionally, in the case of the bearings component (Figure 13b), the air compressor noise (i.e., the most influential feature determined by SHAP diagrams) is very close to the water flow feature. Furthermore, the air compressor noise feature is situated among the least important features in the case of the exhaust valve, water pump, and radiator, suggesting that the conditions of these components are very lightly influenced by the noise present in the air compressor.

**Figure 14:**
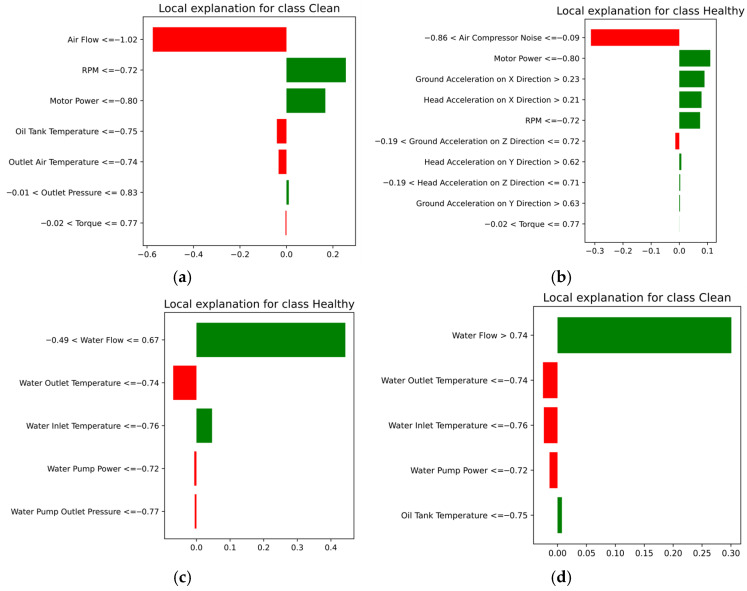
Overview of the local LIME explanation for (**a**) exhaust valve, (**b**) bearing, (**c**) water pump and (**d**) radiator air compressor components. The green bars indicate features that contribute positively toward the predicted class, while the red bars indicate features that contribute negatively.

Figure 14 presents the local LIMEs obtained by the hybrid models of a feature that misclassified two air compressor components (i.e., exhaust valve and bearings) and two features that correctly classified the condition of the water pump and radiator components. For the exhaust valve (i.e., Figure 13a), “Air Flow” and RPM emerged as the most significant factors, where a lower “Air Flow” value substantially reduced the likelihood of a clean classification, while RPM values contributed positively. Similarly, for the bearings (i.e., Figure 14b), air compressor noise, RPM, and ground acceleration in different directions were key indicators; high noise levels negatively impacted the “Healthy” classification, aligning with the mechanical degradation patterns typically observed in faulty bearings. For the water pump (i.e., Figure 14c) LIME diagram, the water flow was the dominant factor, where higher water flow was strongly associated with the “Healthy” condition. Similar trends were noted for the radiator (i.e., Figure 14d), with “Water Flow” and “Water Outlet Temperature” playing critical roles in the model’s decision-making process.

**Figure 15:**
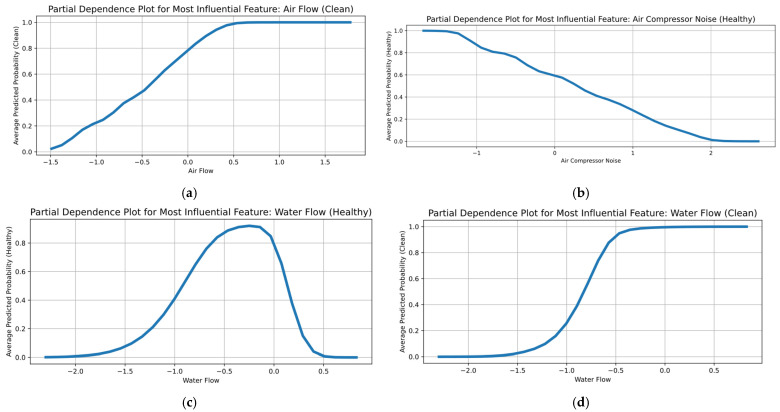
Overview of the PDPs for the (**a**) exhaust valve, (**b**) bearing, (**c**) water pump, and (**d**) radiator air compressor components, corresponding to the “Clean”/“Healthy” class.

Figure 15 shows the PDP diagrams of the hybrid model-based “Clean” class probability model and the most influential feature across the dataset for each air compressor component. Air flow was the most influential feature for the exhaust valve (Figure 15a). When the air flow is very low (i.e., approximately 1.5 ), the model’s likelihood of selecting a “Clean” state is near zero. As air flow increases within the interval [−1.0, +0.5], the probability increases sharply from approximately 0.2 to 0.9, indicating that the model has learned a clear decision boundary between the “Clean” and “Dirty” classes in this interval. Moreover, beyond this threshold, the curve levels off near one. Therefore, after exceeding a critical flow threshold, the model’s estimated probability of the “Clean” condition approaches unity, such that further increases in air flow produce negligible additional gains in confidence. Instead, the air compressor noise (Figure 15b) curve, which is the most influential feature for bearing condition monitoring, reveals a strictly monotonic, negative relationship with the probability of a healthy bearing. At low noise levels, the model is almost certain of a healthy condition. With each increase in noise, the predicted health probability decreases in an almost linear fashion, crossing the decision threshold around the value of 0.5. The confidence of the model in a healthy bearing condition rapidly approaches zero, highlighting that increased acoustic emissions indicate incipient bearing damage.

The PDP curve for the water pump (i.e., Figure 15c) exhibits a clear bell-shape, indicating that pump health is maximized within a specific flow window. Therefore, the likelihood of the pump being classified as “Healthy” increases substantially as flow transitions from sub-optimal, low rates to a defined mid-range optimum, after which further increases in flow lead to a reduction in the model’s estimated health probability. Figure 14d highlights the evolution of the PDP curve characterizing the radiator condition based on the most important feature (i.e., water flow). At very low flow rates, there is a near-zero probability of the model predicting a “Clean” condition. As flow increases through the range [−1.0, −0.5], the probability rises sharply, indicating the critical threshold for adequate coolant circulation. Beyond this threshold, the curve flattens near unity, demonstrating that higher flow reliably predicts a clean radiator.

**Figure 16:**
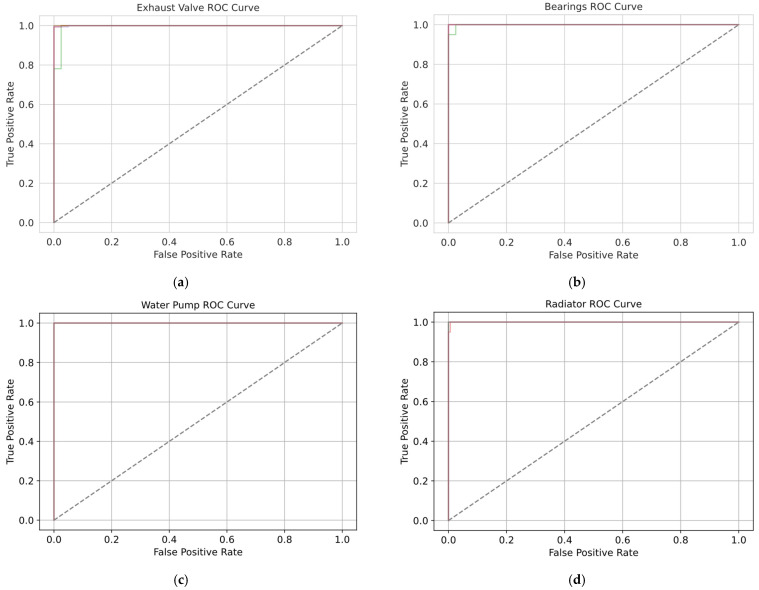
The ROC curves highlighting the hybrid DL-based model’s true positive rate against the false positive rate across each fold for (**a**) exhaust valve, (**b**) bearings, (**c**) water pump and (**d**) radiator components.

The receiver operating characteristic (ROC) curves obtained by the hybrid model for the exhaust valve, bearings, water pump and radiator across all the folds are presented in Figure 16. Across all components and folds, the ROC trajectories ascend sharply from the origin to the upper-left corner and then run almost horizontally, achieving a true positive rate close to the value of one, and yielding AUC values almost equal to unity. Therefore, this behavior demonstrates the effective discriminative capability of the model; it is able to distinguish between healthy and faulty states, achieving true positive rates above 0.99 and false positive rates below 0.01. Moreover, the tight clustering of curves from the five stratified folds indicates minimal variance in performance, underscoring the classifier’s stability and generalizability. Therefore, the proposed hybrid architecture can reliably detect component faults with both high sensitivity and specificity, thereby minimizing missed detections and false alarms in predictive maintenance applications.

**Figure 17:**
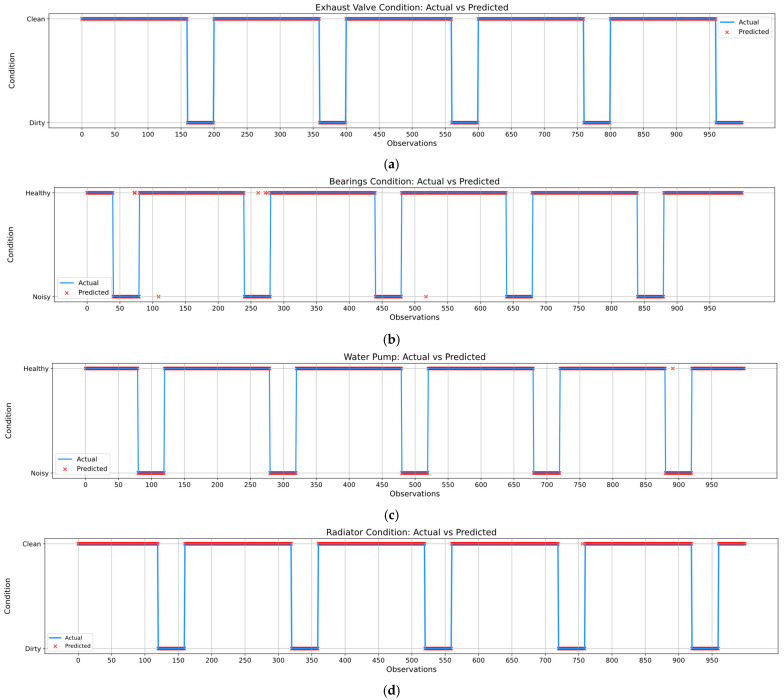
Overview of the actual and predicted values of the hybrid model, which correspond to (**a**) exhaust valve, (**b**) bearings, (**c**) water pump, and (**d**) radiator component of the air compressor.

In order to have a better visual overview of both the actual and the predicted values of each component within the air compressor, Figure 17 shows a side-by-side comparison of the classification results of the hybrid model corresponding to the exhaust valve, bearings, water pump, and radiator, respectively. The plots indicate that the majority of predicted values closely align with the actual values, as seen by the good overlap of red (i.e., predicted) and blue (i.e., actual) markers over the dataset. Therefore, this implies that the DNN-based model can properly identify the condition of each component in the majority of observations. Additionally, Table 10, Table 11, Table 12 and Table 13 highlight the predicted conditions and the corresponding probability, as well as the recommended action that should be taken by the maintenance personnel. However, a few misclassifications can be observed in the case of both the bearings and water pump components. Table 14 presents the misclassified observations corresponding to each component of the air compressor. It can be observed that even though the model misclassified eight observations in the case of the bearings component and one observation in the case of the water pump, the misclassification probability is relatively small, especially in the case of the 109th and 272nd observations.

The integration of the previously mentioned XAI techniques within the decision-making process of DL and ML models, rather than solely relying on the classical EDA, as shown in Figure 6, Figure 7, Figure 8 and Figure 9, highlights a series of advantages for maintenance personnel. On the one hand, SHAP diagrams show both the feature importance and the direction of feature influence per alert. In this way, maintenance personnel can quickly visualize which features are mostly associated with the DL or ML model’s predictions and prune the sensors that are associated with low rank features. Additionally, the LIME method represents a visual justification for case-by-case alarm triggers, showing the feature’s impact of a certain observation. Therefore, it provides an intuitive debugging method for false positive or false negative alarms. Nevertheless, PDP can be a useful technique for maintenance personnel, since it shows the threshold where a certain component changes the state between faulty and non-faulty, thus making them capable of taking informed decisions regarding maintenance scheduling.

### 3.2. Comparison Between a Pure DNN Baseline and a Standalone SVM Model

To compare the difference in terms of performance between the hybrid model and a pure DNN baseline with two hidden layers and a pure SVM model, an ablation across all three devices is presented in Table 15. In terms of overall accuracy, both the DNN and SVM model present slightly reduced yet comparable values across all devices. Additionally, in terms of F1-score, both standalone DNN and SVM models are relatively reduced by 7–9% across all devices compared to the ones obtained by the hybrid architecture. On the other hand, the recall obtained by the SVM models is relatively close to the one obtained by the hybrid model on both NVIDIA Jetson Nano and Raspberry Pi model B devices (i.e., approximately 2%).

**Figure 18:**
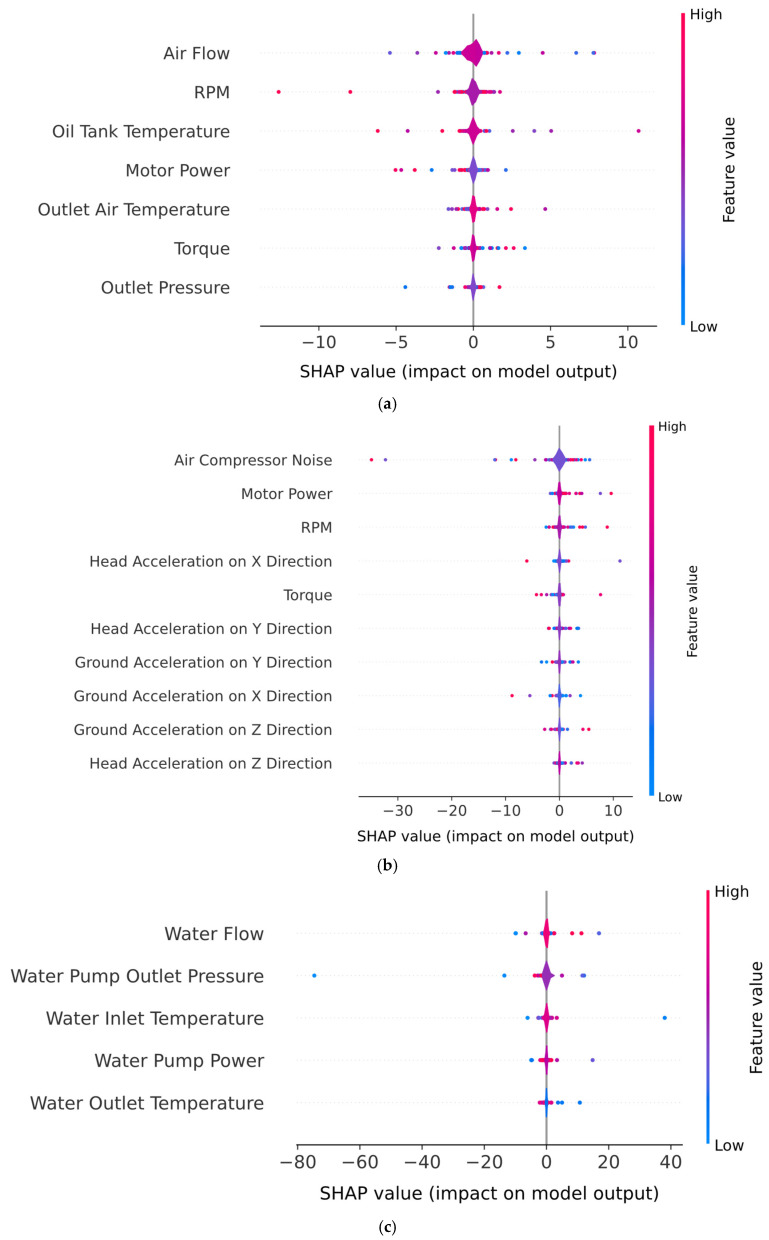

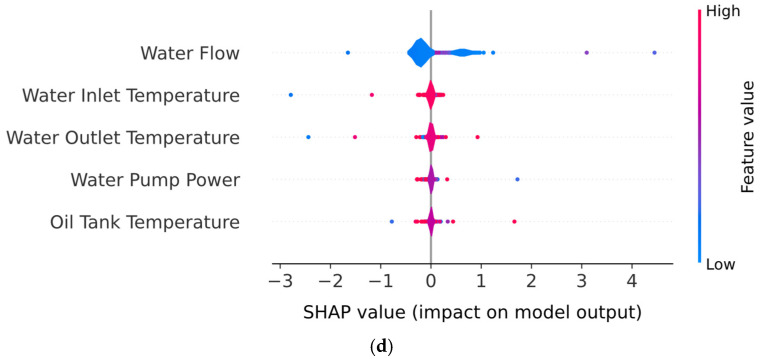
Overview of the beeswarm SHAP diagrams highlighting the contribution of the selected features for (**a**) exhaust valve, (**b**) bearing, (**c**) water pump, and (**d**) radiator air compressor components using a two-layer DNN model.

To highlight the impact of the utilization of a pure DNN baseline on the interpretability of the PdM pipeline, Figure 18 presents the beeswarm diagrams obtained after training and validating a two-layer DNN on every component of the air compressor. It can be clearly observed that the discriminative capabilities of the pure DNN model are reduced compared to the hybrid model across all the components of the air compressor. For instance, in the case of the exhaust valve, bearings, and water pump components, the SHAP values of the top three most influential features are mostly centered around the value of zero, indicating that no clear conclusion can be drawn regarding the impact of these features on the final decision-making process of the pure DNN model. Additionally, the SHAP values of the “Air Flow”, “RPM” (Figure 18a) and “Air Compressor Noise” (Figure 18b) features are represented in purple: that is, medium values. Therefore, no clear conclusion can be made on which features influence the model in terms of the clean/dirty state in the case of the exhaust valve and radiator components, or the healthy/noisy state in the case of the bearings and water pump components. Similarly, in the case of the water pump condition (Figure 18d), the SHAP values are low for both positive and negative SHAP values, indicating that no clear conclusion can be drawn on whether this component is healthy or noisy.

**Figure 19:**
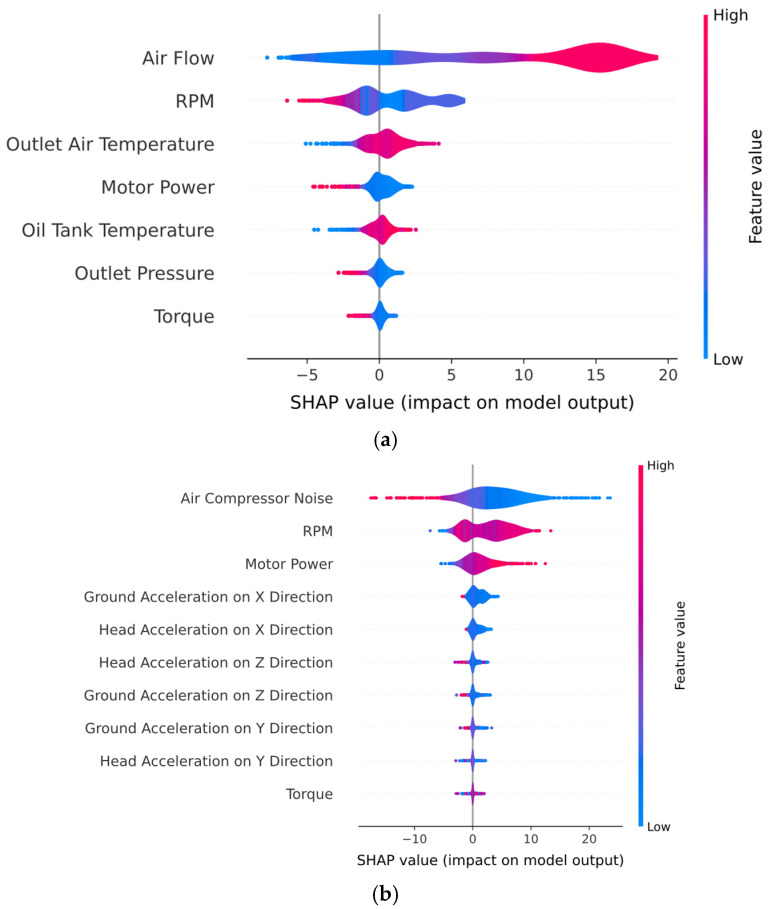

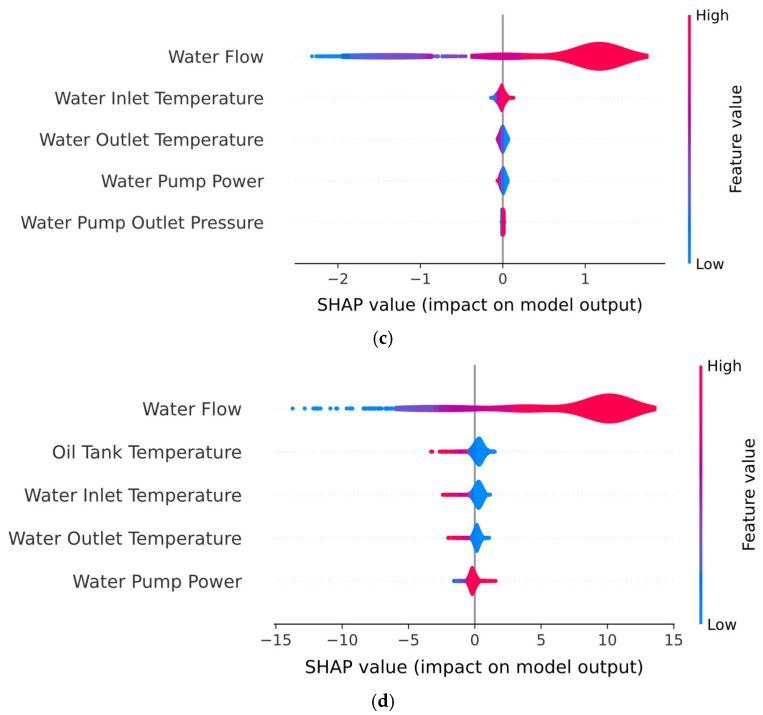
Overview of the beeswarm SHAP diagrams highlighting the contribution of the selected features for the (**a**) exhaust valve, (**b**) bearing, (**c**) water pump, and (**d**) radiator air compressor components, using a standalone SVM model.

Figure 19 presents the beeswarm diagrams obtained after training and validating the standalone SVM model on every component of the air compressor. Compared with the DNN model, the SVM model presents a significantly increased discriminative capability across the entire set of components. For instance, in the case of both exhaust valve (Figure 19a) and bearings (Figure 18b) the boundary between the faulty and non-faulty states is clearly highlighted, especially regarding the air flow and air compressor noise features. However, the SVM model shows reduced discriminative capability for the RPM feature for both the exhaust valve and bearings components. Similarly, in the case of both the water pump (Figure 19a) and the radiator (Figure 19b), the most important feature (i.e., water flow) clearly shows that high SHPA values indicate a healthy state of the components.

Although the model achieved a good performance in condition monitoring across all air compressor components, several limitations of this study should be acknowledged. The main limitation is related to the size of the dataset, which includes 1000 observations. This may, to some extent, limit the model’s ability to fully generalize in production environments, due to the overfitting phenomenon. In fact, when working with small-scale datasets such as the one presented in this paper, even very lightweight models, such as a two-layer DNN, may end up with more trainable parameters (i.e., weights) than the dataset’s degrees of freedom. Additionally, even though five-fold cross validation has been utilized, only a relatively small number of observations are used for validation (i.e., approximately 200 samples). Therefore, a few atypical observations may influence the outcome of the inference results.

Another limitation is the lack of real-world variability in terms of environmental noise or operational disturbances, which could affect the model’s robustness under deployment conditions. Although each observation in the dataset can be clearly visualized in time using line plots, the observations are not associated with explicit timestamps. Consequently, the model does not capture temporal patterns, which may play a role in early fault detection.

## 4. Conclusions

In this paper, a PdM solution for condition monitoring and fault prediction for four principal air compressor components (i.e., exhaust valve, bearings, water pump, and radiator) is compared by using a hybrid architecture, composed of a two-layer and dropout-enhanced DNN model and SVM classifier, a two-layer DNN model, and a standalone SVM model. The hybrid DNN-based feature extractor, followed by an SVM classifier, was trained and validated on two general-purpose devices (e.g., NVIDIA T4 GPU and NVIDIA Jetson Nano) and one resources-constrained device (i.e., Raspberry PI Model B), and reports regarding the training and inference latency, energy consumption, and CO_2_ emissions are presented. To enhance the explainability and transparency of the hybrid model predictions, three model-agnostic XAI techniques were utilized: SHAP, LIME and PDP. Additionally, the impact on interpretability of the pure two-layer DNN and a standalone SVM model has been analyzed using feature importance raking and beeswarm SHAP diagrams. SHAP offers a holistic overview of the importance of selected features for each component, as well as a visual overview of the direction of influence for each feature in the DL-based model. Additionally, PDP helps in identifying how, on average, varying a single feature changes the model’s predicted outcome across the dataset. Finally, LIME generates explanations for individual predictions by locally approximating the behavior of the DL-based model (i.e., illustrating how specific features influence model predictions). The hybrid model achieves on average 98.71%, 99.25%, 98.78%, and 99.01% performance in terms of accuracy, precision, recall, and F1-score across all devices. Additionally, the DNN baseline and SVM model achieves on average 93.2%, 88.33%, 90.45%, and 98.37% as well as 93.34%, 88.11%, 95. 41%, and 91.62% performance in terms of accuracy, precision, recall, and F1-score across all devices. In terms of interpretability, both the hybrid and SVM models clearly differentiate between faulty and non-faulty conditions, whereas the pure DNN architecture presents limited discriminative capability. Although the results obtained by all models are promising, its performance is also influenced by the amount and quality of data that was utilized during the training phase.

Regarding future research directions, we propose utilizing an extended version of this dataset that can also capture temporal information about the air compressor’s degradation state to develop multimodal DL-based models (e.g., using LSTM-based architectures). Additionally, we aim to validate the proposed model under more complex industrial data (i.e., noise and sensor drift phenomena) and integrate it within a real-time industrial monitoring system. Furthermore, we aim to explore other XAI techniques, such as individual conditional expectation (ICE) and Ceteris Paribus Plots, to enhance the transparency and interpretability of DL-based models.

## Figures and Tables

**Table 2:** Hyperparameter settings of both hybrid DL model and DNN model.

Hyperparameter	Value
Learning Rate	10^−3^
Batch Size	256
Activation Function	Leaky ReLU
Loss Function	Binary Cross-Entropy
Optimizer	Adam
Epochs per Fold	7
Exponential Decay Rates	$\beta_{1}=0.8$
	$\beta_{2}=0.999$

**Table 3:** Performance results of the hybrid DL-based model on the NVIDIA T4 GPU.

	Test Accuracy [%]	Precision [%]	Recall [%]	F1 Score [%]
Exhaust Valve	98.4	99.1	98.5	98.3
Bearings	98.6	99.3	98.3	98.6
Water Pump	99.3	99.6	99.4	99.5
Radiator	99.1	99.3	99.5	99.4

**Table 4:** Performance results of the hybrid DL-based model on Raspberry Pi 4 Model B.

	Test Accuracy [%]	Precision [%]	Recall [%]	F1 Score [%]
Exhaust Valve	98.2	98.9	98.5	98.7
Bearings	98.5	98.9	98.4	98.6
Water Pump	99.1	99.5	99.3	99.3
Radiator	99.4	99.3	99.3	99.3

**Table 5:** Performance results of the hybrid DL-based model on the NVIDIA Jetson Nano.

	Test Accuracy [%]	Precision [%]	Recall [%]	F1 Score [%]
Exhaust Valve	98.2	98.9	98.5	98.7
Bearings	98.5	99.4	98.4	98.9
Water Pump	99.1	99.5	99.3	99.3
Radiator	98.2	98.4	98.3	98.3

**Table 6:** Device-level runtime performance recorded on the NVIDIA T4 GPU by the hybrid model.

		Training Time per Epoch [ms]	Inference Time per Epoch [ms]
Exhaust Valve Condition	Fold 1	154.3	63.5
	Fold 2	152.9	61.2
	Fold 3	138.5	61.4
	Fold 4	141.6	62.6
	Fold 5	136.2	62.3
Bearings Condition	Fold 1	149.1	80.1
	Fold 2	151.5	67.8
	Fold 3	145.9	65.2
	Fold 4	149.7	65.8
	Fold 5	136.2	62.3
Water Pump	Fold 1	141.2	58.4
	Fold 2	127.9	59.5
	Fold 3	136.5	63.1
	Fold 4	142.6	62.4
	Fold 5	132.3	63.1
Radiator	Fold 1	137.5	58.2
	Fold 2	122.9	57.3
	Fold 3	126	57.2
	Fold 4	115.7	58.1
	Fold 5	115.2	56.7

**Table 7:** Device-level runtime performance recorded on Raspberry Pi 4 Model B by the hybrid model.

		Training Time per Epoch [ms]	Inference Time per Epoch [ms]
Exhaust Valve Condition	Fold 1	799.6	222.8
	Fold 2	680.5	225.3
	Fold 3	650.1	236.2
	Fold 4	706.4	227.6
	Fold 5	651.3	227.3
Bearings Condition	Fold 1	665.7	218.9
	Fold 2	659.3	220.2
	Fold 3	641.5	233.5
	Fold 4	722.8	230
	Fold 5	678.6	219.1
Water Pump	Fold 1	685.4	223.7
	Fold 2	700.3	225.6
	Fold 3	662.7	222.1
	Fold 4	705.7	223.4
	Fold 5	613.4	224.1
Radiator	Fold 1	686.6	220.3
	Fold 2	627.7	223.1
	Fold 3	643.2	232.6
	Fold 4	673.1	224.6
	Fold 5	642.4	222.2

**Table 8:** Device-level runtime performance recorded on the NVIDIA Jetson Nano by the hybrid model.

		Training Time per Epoch [ms]	Inference Time per Epoch [ms]
Exhaust Valve Condition	Fold 1	497.3	11.9
	Fold 2	523.3	12.9
	Fold 3	501.2	13.6
	Fold 4	535.1	13.4
	Fold 5	586.4	17.3
Bearings Condition	Fold 1	616.7	17.9
	Fold 2	637.4	19.9
	Fold 3	620.8	20.1
	Fold 4	672.4	20.7
	Fold 5	661.2	21.5
Water Pump	Fold 1	691.9	22.2
	Fold 2	706.6	22.9
	Fold 3	724.7	24.5
	Fold 4	739.1	24.7
	Fold 5	73.51	25.4
Radiator	Fold 1	708.5	26.4
	Fold 2	799.8	26.6
	Fold 3	785.8	27.3
	Fold 4	805.9	28.2
	Fold 5	819.6	28.9

**Table 9:** Memory usage, energy consumption, and CO_2_ emissions recorded on NVIDIA T4 GPU, NVIDIA Jetson Nano and Raspberry Pi Model B by the hybrid model.

		Memory Usage [Mb]	Energy Consumption [Wh]	CO_2_ Emissions [g]
NVIDIA T4 GPU	Exhaust Valve	2073.8	1.59	5.56
	Bearings	2082.2	1.68	4.81
	Water Pump	2078.9	1.67	4.76
	Radiator	2073.5	1.63	4.67
NVIDIA Jetson Nano	Exhaust Valve	1472.4	0.59	1.51
	Bearings	1473.1	0.60	1.53
	Water Pump	1472.9	1.10	2.81
	Radiator	1477.2	0.71	1.87
Raspberry Pi 4 Model B	Exhaust Valve	705.7	0.13	0.033
	Bearings	688.2	0.12	0.091
	Water Pump	701.5	0.13	0.032
	Radiator	704.3	0.13	0.037

**Table 10:** Predicted condition, probability of failure, and recommended operational actions for the exhaust valve, based on observation intervals.

Observations	Predicted Condition	Probability	Recommended Action
0–159	Clean	98.64%	Monitoring
160–200	Dirty	98.53%	Cleaning
201–359	Clean	98.38%	Monitoring
360–400	Dirty	99.1%	Cleaning
401–559	Clean	98.79%	Monitoring
560–599	Dirty	98.51%	Cleaning
600–759	Clean	98.47%	Monitoring
760–800	Dirty	99.12%	Cleaning
801–959	Clean	98.24%	Monitoring
960–1000	Dirty	98.49%	Cleaning

**Table 11:** Predicted condition, probability of failure, and recommended operational actions for the bearings, based on observation intervals.

Observations	Predicted Condition	Probability	Recommended Action
0–39	Healthy	98.67%	Monitoring
40–79	Noisy	98.65%	Schedule Maintenance
80–239	Healthy	99.07%	Monitoring
240–279	Noisy	98.53%	Schedule Maintenance
280–439	Healthy	98.71%	Monitoring
440–479	Noisy	98.63%	Schedule Maintenance
480–639	Healthy	98.11%	Monitoring
640–679	Noisy	98.23%	Schedule Maintenance
680–839	Healthy	98.55%	Monitoring
840–879	Noisy	98.52%	Schedule Maintenance
880–1000	Healthy	99.31%	Monitoring

**Table 12:** Predicted condition, probability of failure, and recommended operational actions for the water pump, based on observation intervals.

Observations	Predicted Condition	Probability	Recommended Action
0–79	Healthy	99.21%	Monitoring
80–119	Noisy	98.65%	Schedule Maintenance
120–279	Healthy	98.27%	Monitoring
280–319	Noisy	98.31%	Schedule Maintenance
320–479	Healthy	98.42%	Monitoring
480–519	Noisy	98.23%	Schedule Maintenance
520–679	Healthy	98.55%	Monitoring
680–719	Noisy	98.51%	Schedule Maintenance
720–879	Healthy	98.48%	Monitoring
880–919	Noisy	98.57%	Schedule Maintenance
920–100	Healthy	98.32%	Monitoring

**Table 13:** Predicted condition, probability of failure, and recommended operational actions for the radiator, based on observation intervals.

Observations	Predicted Condition	Probability	Recommended Action
0–119	Clean	98.32%	Monitoring
120–159	Dirty	99.12%	Cleaning
160–319	Clean	98.45%	Monitoring
320–359	Dirty	98.55%	Cleaning
360–519	Clean	98.49%	Monitoring
520–559	Dirty	98.59%	Cleaning
560–719	Clean	99.06%	Monitoring
720–759	Dirty	98.73%	Cleaning
760–919	Clean	98.52%	Monitoring
920–959	Dirty	98.44%	Cleaning
960–1000	Clean	98.51%	Monitoring

**Table 14:** Misclassified observations across the components of the air compressor.

Component	Observations	Predicted Condition	Actual Condition	Probability
Bearings	72	Healthy	Noisy	95%
	73	Healthy	Noisy	90%
	109	Noisy	Healthy	65%
	261	Healthy	Noisy	97%
	272	Healthy	Noisy	76%
	275	Healthy	Noisy	94%
	517	Healthy	Noisy	88%
	679	Noisy	Healthy	94%
Water Pump	891	Healthy	Dirty	86%

**Table 15:** Ablation study against pure DNN baseline with two hidden layers and a standalone SVM model.

			Test Accuracy [%]	Precision [%]	Recall [%]	F1-Score [%]
Two layer DNN with 32 and 16 neurons, respectively	NVIDIA T4 GPU	Exhaust Valve	95.4	91.2	94.6	92.8
		Bearings	93.8	92.1	84.5	88.1
		Water Pump	90.5	85.2	83.2	84.2
		Radiator	95.7	92.3	93.4	92.8
	NVIDIA Jetson Nano	Exhaust Valve	94.3	88.6	95.2	91.7
		Bearings	89.4	82.5	82.4	82.4
		Water Pump	93.1	87.3	91.4	89.3
		Radiator	92.5	86.4	92.2	89.3
	Raspberry Pi 4 Model B	Exhaust Valve	95.7	91.2	97.4	94.1
		Bearings	88.3	81.2	84.7	82.9
		Water Pump	92.6	86.3	90.1	88.1
		Radiator	97.2	95.7	96.4	96.1
SVM Model	NVIDIA T4 GPU	Exhaust Valve	91.3	84.5	94.3	89.1
		Bearings	97.1	95.1	94.2	94.6
		Water Pump	89.4	82.3	93.4	87.4
		Radiator	96.5	93.6	98.1	95.7
	NVIDIA Jetson Nano	Exhaust Valve	92.6	85.4	95.2	88.7
		Bearings	95.2	90.2	97.6	93.7
		Water Pump	88.7	81.3	93.5	86.9
		Radiator	97.5	94.4	97.2	95.7
	Raspberry Pi 4 Model B	Exhaust Valve	91.3	84.1	94.7	89.1
		Bearings	95.6	90.3	97.2	93.6
		Water Pump	88.1	81.2	93.1	86.7
		Radiator	96.8	94.7	96.5	95.6

## Data Availability

The original data presented in the study are openly available at: https://www.kaggle.com/datasets/afumetto/predictive-maintenance-dataset-air-compressor/ (accessed on 13 September 2025).

## Abbreviations

The following abbreviations are used in this manuscript:

ADASYN	Adaptive Synthetic Oversampling
CPS	Cyber-Physical Systems
DL	Deep Learning
DNN	Deep Neural Network
EDA	Exploratory Data Analysis
GPU	Graphical Processing Unit
HVAC	Heating, Ventilation and Air Conditioning
LIME	Local Interpretable Model-agnostic Explanation
ML	Machine Learning
PdM	Predictive Maintenance
PDP	Partial Dependence Plot
SHAP	Shapley Additive Explanation
SVM	Support Vector Machines
XAI	Explainable AI
